# High Molecular Conductance and Inverted Conductance Decay over 3 nm in Aminium-Terminated Carbon-Bridged Oligophenylene-Vinylenes

**DOI:** 10.1021/jacs.4c13901

**Published:** 2024-12-20

**Authors:** Luisa K. I. Rieger, Susanne Leitherer, William Bro-Jørgensen, Gemma C. Solomon, Rainer F. Winter

**Affiliations:** † Department of Chemistry, University of Konstanz, 78434 Konstanz, Germany; ‡ Nano-Science Center and Department of Chemistry, University of Copenhagen, DK-2100 Copenhagen, Denmark; § NNF Quantum Computing Programme, Niels Bohr Institute, University of Copenhagen, DK-2100 Copenhagen, Denmark

## Abstract

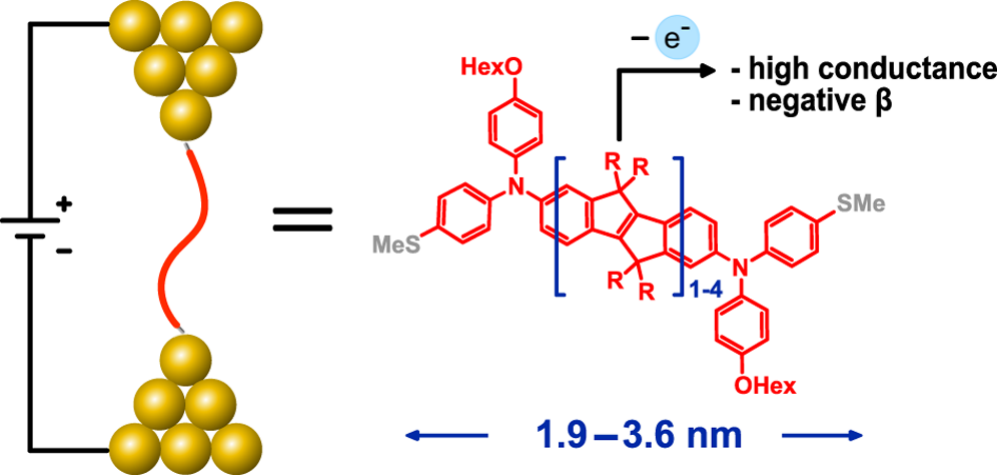

With the progressing miniaturization of electronic device components to improve circuit density while retaining or even reducing spatial requirements, single molecules employed as electric components define the lower limit of accessible structural width. To circumvent the typical exponential conductance decay for increasing length in molecule-based wires, topological states, which describe the occurrence of discontinuities of a bulk material’s electronic structure confined to its surface, can be realized for molecules by the introduction of unpaired spins at the molecular termini. The resulting high conductance and reversed conductance decay are typically only observed for shorter molecules, as the terminal spins must be within the electronic coupling range to produce the desired effects. We expand the realm of long and exceptionally conductive molecular wires by employing highly conjugated, planarized carbon-bridged oligo(phenylene-vinylene)s as conduits between readily oxidizable diarylamine termini. This yields molecular wires of already decent conductance values and small conductance decay in the neutral state. Upon the introduction of topological states, the conductance can be increased by a factor of up to 1800 for a 3 nm long molecule, and the conductance decay becomes inverted, together with an excellent signal intensity at concentrations as low as 0.01 mM.

## Introduction

**Figure 1:**
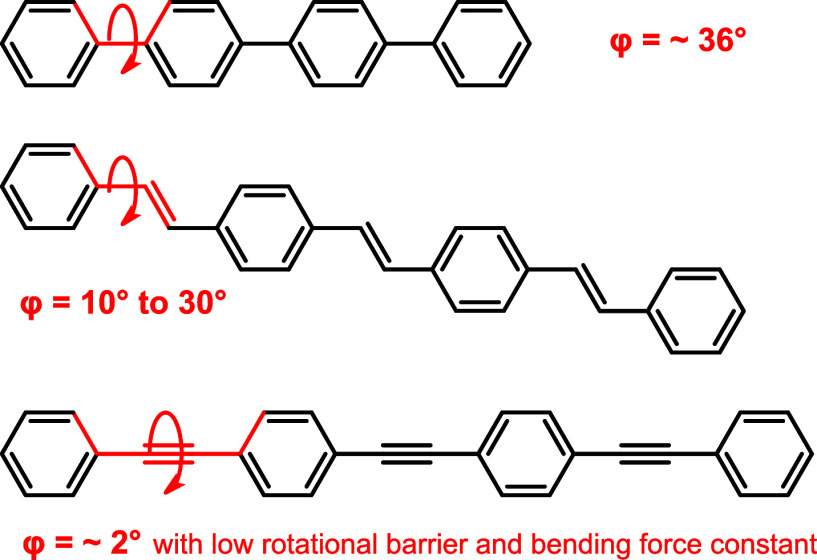
Dihedral angles typically observed in oligo *p*-phenylenes,^28,29^*p*-phenylene-vinylenes,^30^ and *p*-phenyleneethynylenes.^29,31^

The realm of molecular electronics holds the prospect of downsizing integrated logical circuits to the ultimate limit of individual molecules. This comes with the requirement of an efficient wiring with no loss of energy and information on the electron phase. While ballistic, electron transport through a molecular wire usually suffers from an exponential decay with increasing molecular dimensions according to the expression *T* = *A* exp(−β *l*), where *T* is the transmission, *A* a pre-exponential coefficient, *l* the wire length, and β the so-called attenuation or decay factor. In efforts to minimize β, researchers have resorted to the superior transport performances inherent to π-conjugated molecular backbones, with oligophenylenes (OPs),^1−4^ oligophenylene-ethynylenes (OPEs)^5−11^ and oligophenylene-vinylenes (OPVs)^12,13^ ranking among the best-performing motifs. Despite efficient conjugation, the β values of OPEs and OPVs with thiolate or thioether anchor groups typically range around 0.17 Å^–1^, where OPVs perform slightly better than OPEs^5,9,14−16^ (note that a β value of 0.05 Å^–1^ has been reported for dithiocarboxylate-terminated OPEs^4^). Different strategies have been proposed^17−20^ and tested to achieve the ideal limit of β approaching zero or even assuming negative values, indicating efficient charge transport independent of the molecular wire dimension, or even conductance increasing with molecular lengths.^21^ Among them is extension of the π-conjugated system by the insertion of additional π-conjugated repeating units, thereby closing the HOMO–LUMO gap and bringing the molecular orbitals in closer energetic proximity to the electrodes’ Fermi level. This is however counteracted by the lowering of the orbital coefficients at the contacting sites, as well as by increased overall molecular distortion. Bending or twisting of individual wire segments leads to damping as is, e.g., indicated by the scaling of conductance across biphenylene or OPE wires with cos^2^φ (see Figure 1),^1,22,23^ or the increase of molecular conductances across OPs at higher temperature due to a higher population of more planarized structures.^2^ Nevertheless, very small attenuation factors were realized for a series of polyacenes containing ring-fused dibenzothiophene building blocks within their rigid, π-conjugated backbone,^24^ nanoribbons of (edge-)fused porphyrins,^25,26^ or polymethines with an odd number of sp^2^ carbon centers and small bond length alternation along the π-conjugated backbone.^27^ Guided by topological insulators (TIs) from semiconductor physics, researchers have recently succeeded in designing molecular wires with β < 0, by introducing radical centers as so-called topological (edge) states. In solid state physics, edge states describe discontinuities of a bulk material’s electronic structure. At the molecular level, they can be introduced as paramagnetic, oxidized centers with an unpaired spin at one or both termini.^21,32,33^ As oxidized molecules with open-shell topological states are far better conducting than those in the corresponding neutral, closed-shell state, these wires also act as efficient redox-triggered conductance switches. In the present work, we have combined the two approaches from above by utilizing rigidified, planarized carbon-bridged OPVs, so-called COPVs,^34^ with 1 to 4 repeat units (COPV1–4) as the molecular bridges between two anchor group-modified triarylamines. The resulting SMeDACOPVs are easily oxidized to persistent, open-shell triarylaminium radicals.^35^ While backbone stiffening already results in an exceptionally small β value of 0.05 Å^–1^ for the neutral COPVs, amine oxidation boosts conductances by more than 2 orders of magnitude and further diminishes β to slightly below zero.

## Results and Discussion

**Scheme 1:**
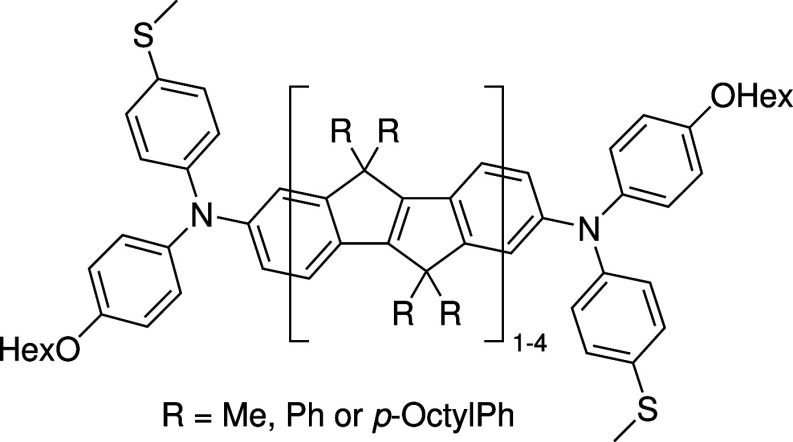
Structure of SMeDACOPV1–4

The series of anchor group-modified diarylamine-capped COPVs (Scheme 1) featuring one to four repeating units (SMeDACOPV1–4) were synthesized as reported in the literature^34,35^ with minor modifications. All molecules are characterized by NMR and UV–vis/NIR spectroscopy. Their singly and doubly oxidized forms were obtained by oxidation with 1 or 2 equiv of silver hexafluoroantimonate respectively and characterized by UV–vis/NIR spectroscopy. Procedures and spectra are provided in the Supporting Information (SI).

Thiomethyl anchoring groups, which reliably bind to surface Au atoms of the nanoelectrodes to form molecular junctions, are situated at one of the *p*-phenylene positions of each diarylamine headgroup. Varying substitution patterns (R = Me, Ph, *p*-octyl-Ph) at the bridging carbon atoms of the COPV bridges are not expected to affect the conductance path, as σ-conductance channel contributions are negligible in the presence of a π-channel,^36,37^ and the latter should be largely unaffected by substitution of the saturated neighbor atom. This is confirmed by no significant difference in transport functions for calculations performed with side groups included compared to side group truncation to methyl units (see Figure S19 in the SI). The series comprises congeners with molecular lengths of 1.9–3.6 nm as measured by the S–S distance with radical center distances of 1.2–3.0 nm, as judged from the N–N distance (see Table 1).

**Table 1:** Total Lengths (S–S) and Radical Center Distances (N–N) of SMeDACOPV*n* Obtained from Theoretical Calculations

*n*	S–S (nm)	N–N (nm)
1	1.9	1.2
2	2.4	1.8
3	3.0	2.4
4	3.6	3.0

### Molecular Conductance Measurements

Molecular conductance measurements were performed using the STM-BJ method with gold electrodes according to literature procedures.^1,37−40^ Histograms were plotted from >5000 individual traces unless stated otherwise. Details are provided in the SI.

#### Neutral Forms

**Figure 2:**
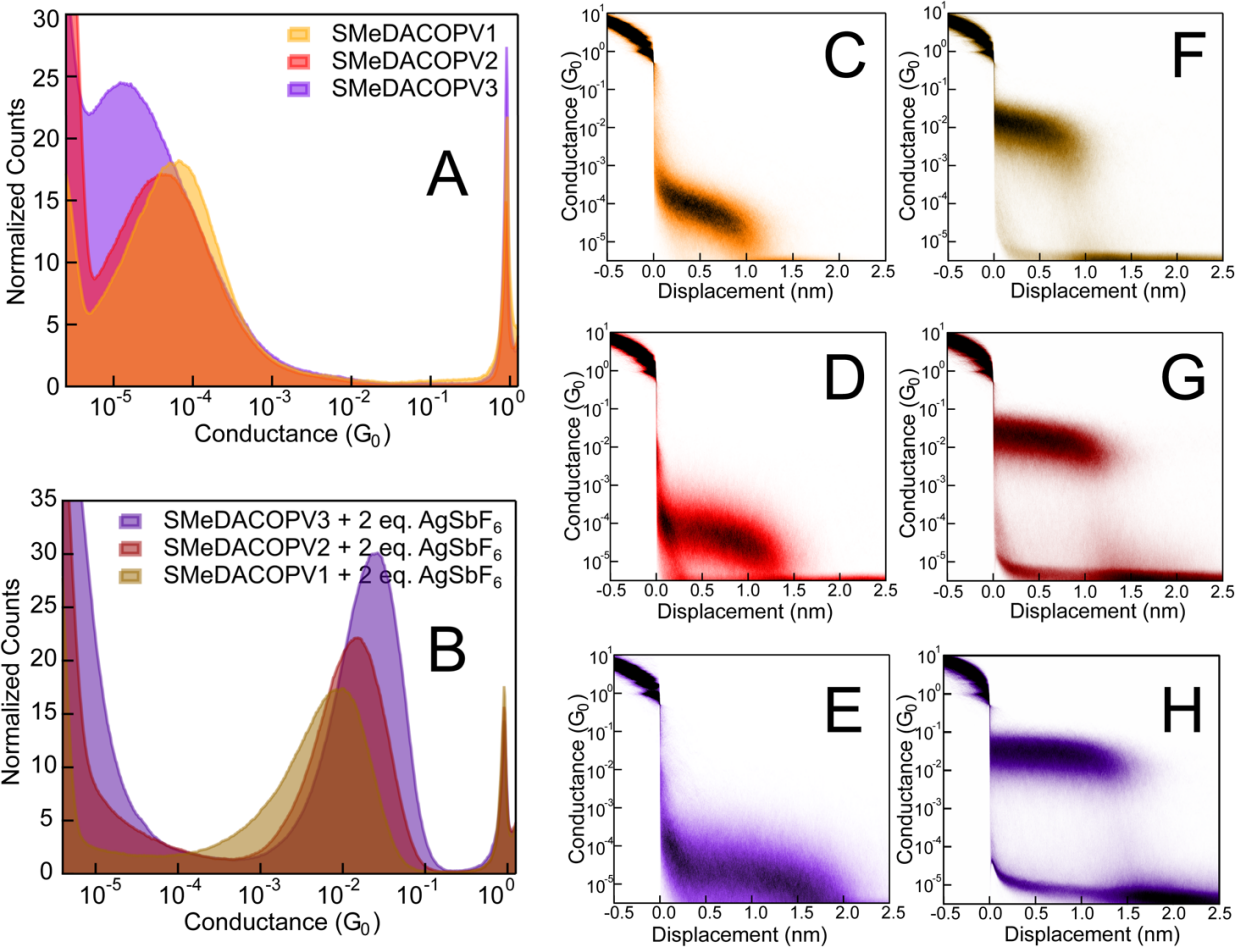
1D conductance histograms obtained for the series of SMeDACOPV1–3 in their neutral (A) and dioxidized (B) forms at *V* = 100 mV. The respective 2D histograms of the neutral compounds are depicted in figures C–E, and of the dioxidized compounds in F–H.

Figure 2 depicts conductance data of the neutral forms (panel A) measured in 1,2,4-trichlorobenzene (TCB) at 100 mV bias voltage, with Zn dust added to prevent oxidation. The neutral oligomers SMeDACOPV1–3 exhibit well-defined monomodal molecular conductance features peaking within the range of 10^–5^ G_0_. No molecular features in this range were however observed for SMeDACOPV4. The 2D histograms show rupture lengths that, after considering the snap back of 0.5 nm that accounts for an initial opening of the nanogap of 0.5 nm at the beginning of the tip excursion of this setup,^41,42^ are slightly shorter than the molecular anchor group distances (see Table 1). This likely indicates a tilted alignment (∼ 45°) during the course of junction elongation, with contact rupture occurring before the molecule is fully erected. The conductance feature of SMeDACOPV3 is partly convoluted with the electrical noise starting at around 5.5 × 10^–5^ G_0_, possibly artificially extending the feature flank and consequently the apparent rupture length.

**Figure 3:**
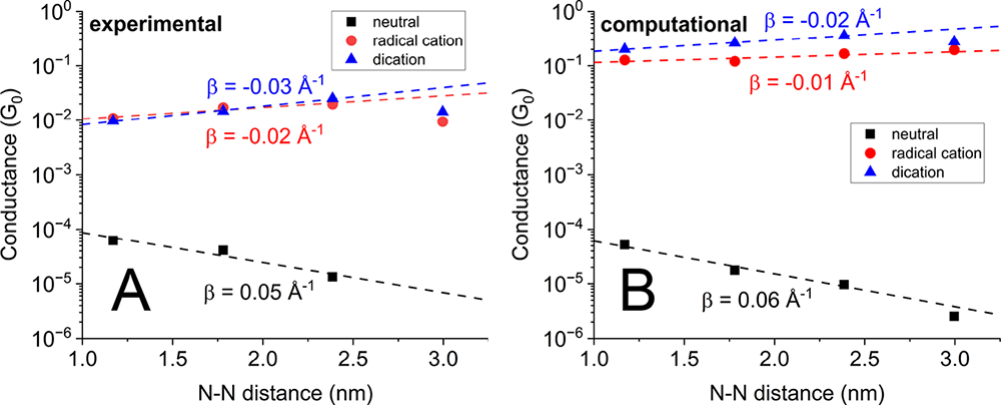
(A) Conductance values obtained for measurements following application of the neutral (black squares), singly (red dots) and doubly (blue triangles) oxidized forms of SMeDACOPV1–4 plotted on a semilogarithmic scale against the N–N distance. No signature was obtained for neutral SMeDACOPV4 under the described conditions. Apparent linear fits of the data points for SMeDACOPV1–3 are used to extract the β parameters. When including SMeDACOPV4, β-parameters of close to zero (singly oxidized) and −0.01 Å^–1^ (doubly oxidized) are obtained. (B) Computationally derived conductance values and the resulting β parameters. Data points for SMeDACOPV4 were omitted from the linear fit for comparability with the experimental results. When including SMeDACOPV4, β-parameters of −0.01 Å^–1^ (singly oxidized) and close to zero (doubly oxidized) are obtained.

Figure 3A provides a semilogarithmic plot of the conductance values of SMeDACOPV1–3 against the molecular N–N distance. The corresponding linear fit, with its slope quantified by the attenuation factor β, represents the dependence of conductance on the molecular length according to *T* = exp(−β·*l*), with *T* and *l* corresponding to the transmission probability and transmission path length, respectively. For the series of neutral molecules, a modest conductance decay of 0.05 Å^–1^ is observed. This represents an improvement as compared to conventional, conformationally unrestricted OPVs of similar length range, whose β value corresponds to ca. 0.17 Å^–1^.^12^ As a result, the conductance of the trimer SMeDACOPV3 falls within the same order of magnitude as that of the monomer SMeDACOPV1 in spite of an increase of molecular length from 1.9 to 3.0 nm. This proves the suitability of COPVs as components of molecular wires that exhibit an exceptionally small conductance decay.

#### Oxidized Forms

**Figure 4:**
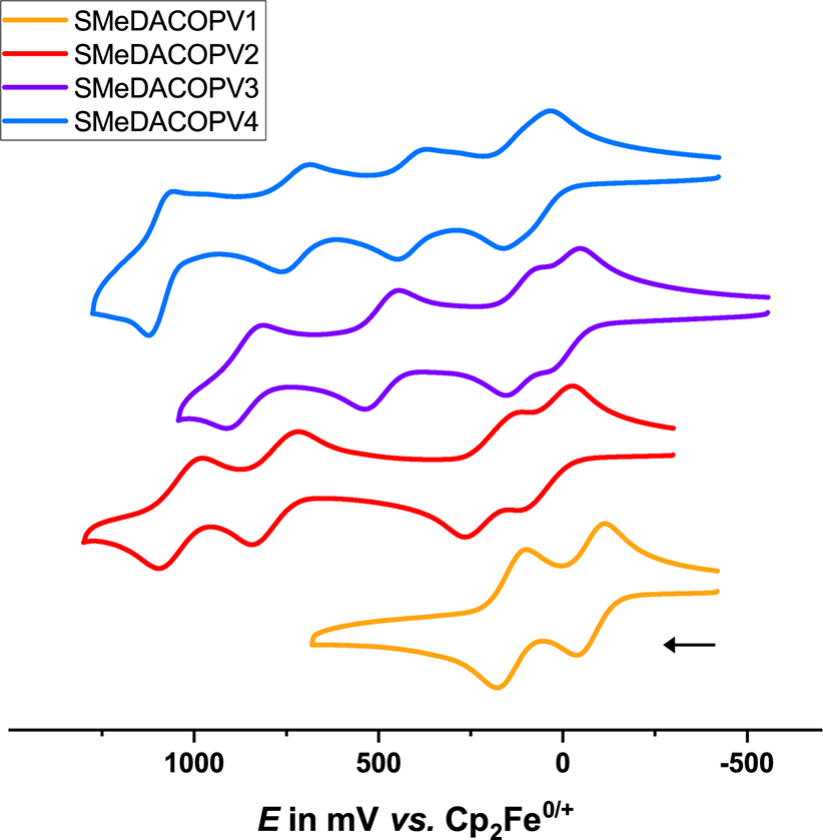
Cyclic voltammograms of the SMeDACOPVs measured in DCM with a glassy carbon working electrode and 0.1 M TBAPF_6_ as the electrolyte.

Like other bridged bis(triarylamines) with π-conjugated linkers, SMeDACOPV1–4 undergo two successive one-electron oxidations at their termini (Figure 4). The half-wave potentials for amine oxidations as determined by cyclic voltammetry of millimolar solutions in 0.1 M NBu_4_^+^PF_6_^–^ (TBAPF_6_) fall below 250 mV on the ferrocene/ferrocenium scale, which signifies the comparable ease at which the topological edge states are generated. As was observed for the closely related series of DACOPVs, which differ from SMeDACOPVs only by the absence of a thiomethyl anchoring group,^35^ the half-wave potential splittings between the second and the first oxidation become smaller as the radical–radical distance increases. This renders the singly oxidized forms of particularly the longer congeners of this series susceptible to disproportionation into the parent neutral and the doubly oxidized forms. Starting with SMeDACOV2, two or three additional COPV-based oxidations become observable at higher potentials, which shift to lower potentials as the COPV backbone gets more expanded. SMeDACOPV1 only shows irreversible processes at higher potentials. These higher oxidations are however of no relevance for our conductance studies.

The oxidized counterparts of SMeDACOPVs1–4 were synthesized and isolated as radical cations and dications, respectively, and their performance as molecular wires was explored under identical conditions to those employed for their neutral congeners. Figure 2B shows 1D conductance histograms of the dioxidized diradicals SMeDACOPV1–3^2+^; histograms of the radical cations are attached in the SI. Oxidation to cationic states causes a drastic increase of conductance compared to the neutral analogs, with conductance distributions peaking in the range of 10^–2^ G_0_. The molecular features are monomodal and of high intensity, resulting in excellent signal-to-noise ratios. The 2D histograms show rupture lengths that correspond with those of their neutral analogues (see Figure 2F,G), yet rupture lengths are better defined due to the clearer differentiation of molecular conductance features from the noise background. Figure 3 reveals that the conductance decay with molecular length becomes indeed inverted for the oxidized species, with a β parameter of −0.02 to −0.03 Å^–1^. The latter values are less negative compared to that of −0.21 Å^–1^ for the singly, and that of −0.07 Å^–1^ for the doubly oxidized forms of nonrigidified oligophenylene-bridged bis(diaryl amines). This together with the small attenuation factor observed for the neutral series demonstrates that the conductance behavior of COPV bridges is overall less sensitive to changes of molecular lengths, substantiating their suitability for molecular wires. Length-independent conducting wires could be of particular benefit for the fabrication of molecular devices for practical application, as the junction microenvironment of several types of well performing molecule–electrode interfaces proves to be a challenging factor to control. Ensuing difficulties in achieving well-defined, uniform gap sizes^43^ cause reproducibility issues, such as in the case of graphene nanojunctions.^44−47^ A mixture of molecular wires of uniform conductance, but different dimensions to accommodate varying gap sizes, could be an elegant solution to this issue.

Figure 3 reveals almost no difference in conductance between the mono- and dioxidized cationic species, with the observed variations falling within the inherent error limits to be expected between individual measurements. This might indicate that the one- and two-electron oxidized forms interconvert under the given experimental conditions, which is supported by the generally modest potential splitting (see Table T2 of the SI) of the two redox processes revealed by cyclic voltammetry (see Figure 4). Further indication for possible redox state interconversion during the measurement is provided by the fact that the radical cation of the monomer SMeDACOPV1, which shows the largest redox wave splitting of all congeners, disproportionates partially when dissolved in TCB, the solvent employed in our experiments (see Figure S10 in the SI). The decreasing potential splitting of the higher oligomers should enhance the likeliness of disproportionation further. Additionally, a change in charge state might be facilitated by the applied bias voltage, which could match the relatively small oxidation potentials of the SMeDACOPVs. It should however be mentioned that the data are collected from many individual single molecule binding events where the molecule is far from its equilibrium state, experiencing conditions that greatly differ from those present in bulk solutions. Comparisons between individual molecules embedded within a molecule junction and ensembles of molecules in bulk solution must therefore be made with great caution. Given the very similar conductance values of the chemically generated singly and doubly oxidized SMeDACOPV1–3, we cannot exclude that the experimentally observed conductance distributions correspond to a superposition of mono- and dications confined within the molecular junctions. This is supported by our transport calculations, which reveal only minor differences in conductance values obtained from cations and dications (see below).

**Figure 5:**
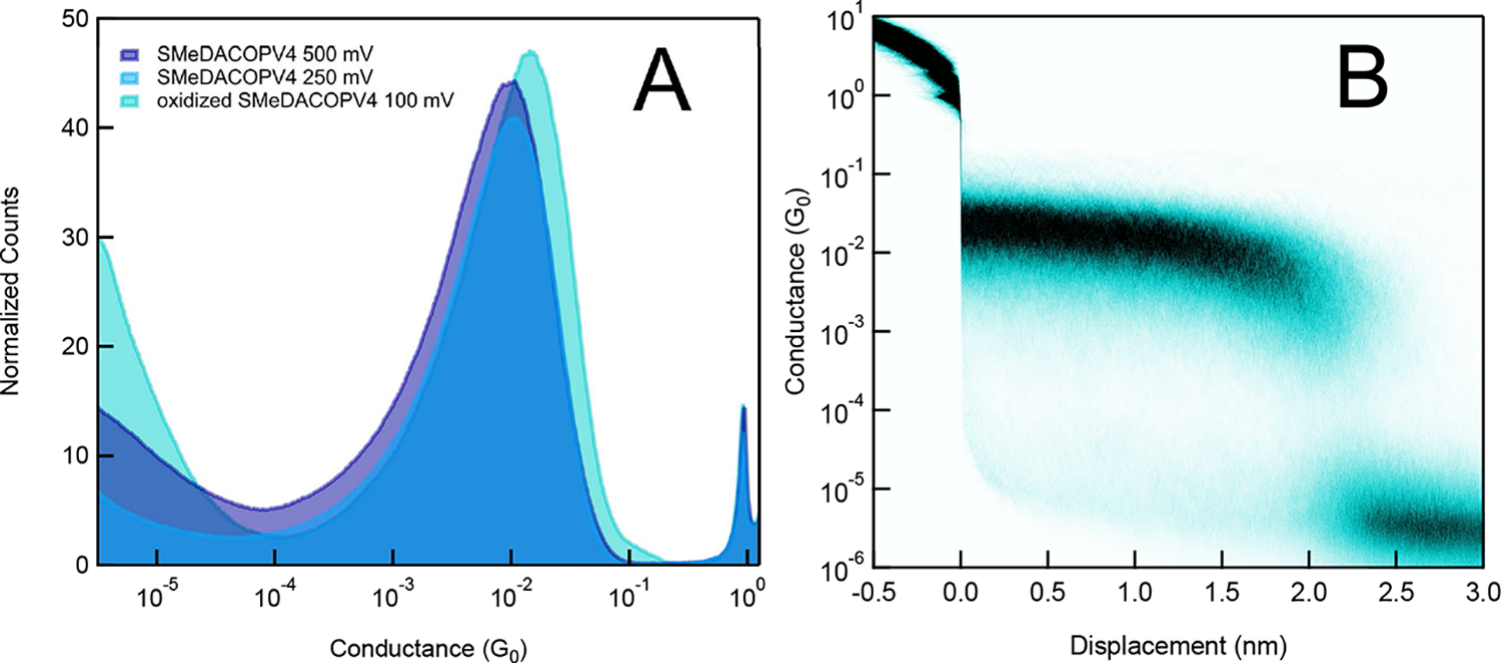
1D and 2D conductance histograms for oxidized SMeDACOPV4 at 100 mV and for SMeDACOPV4 oxidized in situ by applying a bias voltage *V* of 250 and 500 mV (A). The 2D histogram (B) corresponds to the measurement at *V* = 100 mV.

Chemically oxidized SMeDACOPV4 was found to be unstable over prolonged measurement times at a bias voltage of 100 mV, which allowed for only a limited number of traces to be recorded before significant conductance value changes ensued. 1D histograms compiled of the initial 3000 traces display conductance values of around 1 × 10^–2^ G_0_, which is slightly lower than the values acquired for SMeDACOPV3, thus breaking the trend of inverted conductance decay. This aligns with previous studies^33^ and is the result of a loss of spin coupling/redox center communication with increasing distance. The conductance of SMeDACOPV4^1+/2+^ is close to the value derived from theoretical calculations. The latter also predict that doubly oxidized SMeDACOPV4 performs slightly worse than its shorter congener SMeDACOPV3^2+^ (see below). Measurements on neutral SMeDACOPV4 at *V* = 250 and 500 mV reveal a stable molecular feature with similar conductance values (see Figure 5), indicating that oxidized SMeDACOPV4^*n*+^ (*n* = 1 or 2) can be obtained in situ from the neutral compound upon application of a sufficiently high bias voltage. It furthermore shows that redox processes readily occur for this type of compound during STM-BJ measurements (vide supra). We note that similar results were obtained when studying in situ oxidation of SMeDACOPV3 at bias voltages between 100 and 750 mV (see SI). The 2D histogram indicates a rupture length of approximately 2.7 nm with consideration of the snap back correction, which is again substantially shorter than the molecular S–S distance of 3.6 nm. Assuming premature contact rupture, this would correspond to a maximum erection of the molecule by 46° within the junction.

### Theoretical Calculations

To analyze the experimental results, we conducted transport calculations for molecular junctions featuring the SMeDACOPV1–4 molecules in their neutral, singly and doubly oxidized states. These calculations employed density functional theory (DFT) via the SIESTA code,^48^ in combination with the nonequilibrium Green’s function (NEGF) method (for further details, please refer to the SI).

Initially, the isolated molecules were optimized in vacuum and subsequently connected through the S atoms to simple model electrodes, which were described using the wide-band approximation. Test calculations involving the neutral SMeDACOPV1, which included multiple layers of gold as electrodes in the transport models, indicated a low level of hybridization between the molecular orbitals and those of the gold electrodes. This finding is consistent with previous studies of TI molecules linked to gold electrodes, where both weak coupling and low hybridization between molecular orbitals and the electrode were observed, even in charged species.^33^ We discovered that explicitly incorporating gold electrodes can significantly influence the alignment of molecular resonances with the electrode Fermi energy. However, this inclusion does not notably alter the relative energetic positions of the resonances, and the trends observed in this study remain consistent (see the discussion in the SI, Figure S18). As the side groups have minimal impact on transmission (see SI Figure S19), we have replaced all side groups with methyl groups for simplicity.

**Figure 6:**
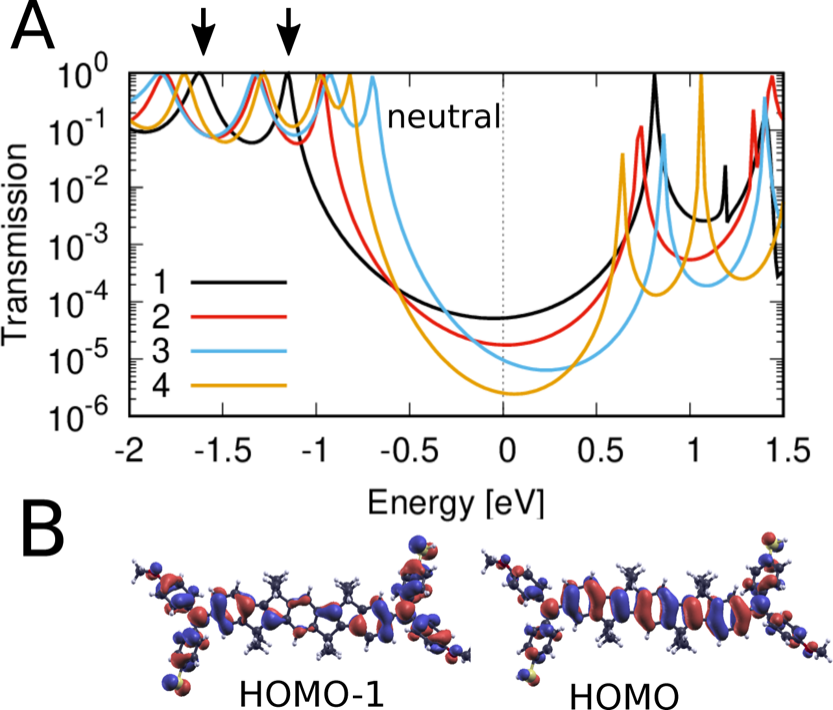
(a) Transmission through the neutral species of SMeDACOPV*n*, with *n* = 1, 2, 3, and 4. The transmission at the Fermi level diminishes as the molecular length increases, moving from *n* = 1 to *n* = 4. (b) Highest occupied molecular orbitals HOMO and HOMO–1 of SMeDACOPV2, dominating the transport at *E*_F_.

The transmission through the neutral SMeDACOPV1–4 are depicted in Figure 6A. Notably, the lowest unoccupied molecular orbital (LUMO) exhibits minimal coupling to the electrodes, as evidenced by its narrow transmission resonances and low orbital density on the S atoms (see SI, Figure S17). In contrast, the transmission at the Fermi level is predominantly influenced by resonances associated with the two highest occupied molecular orbital levels HOMO and HOMO–1. As the length of the SMeDACOPV1–4 molecules increases, the energies of the HOMO and HOMO–1, which are illustrated for SMeDACOPV2 in Figure 6B, converge more closely. This trend suggests a reduction in the end-to-end electronic coupling through the molecule, particularly through the π orbitals.^49^ As a result, the transmission at the Fermi level diminishes with increasing molecular length. We note that the HOMO resonance of SMeDACOPV4 is lower than that of SMeDACOPV3, which we attribute to a slight bending in SMeDACOPV4 resulting from its increased length. These subtle differences might also affect the Fermi energy, as calculated by the Siesta code (see Methods section in SI). Most notably, the trend of decreasing transmission from *n* = 1 to *n* = 4 is generally observed across an energy range of 1 eV surrounding *E*_F_. The corresponding exponentially decreasing conductance of SMeDACOPV1–4, derived from *G* = *G*_0_*T*(*E*_F_), closely aligns with experimental findings, as illustrated in Figure 3.

To simulate the monocation and dication forms of SMeDACOPV1–4, we increased the net charge by +1 and +2, respectively, and incorporated spin-polarization in our DFT calculations. It is important to note that the monocation/dication species are characterized as open-shell doublets/open-shell singlets, which can pose challenges for accurate description using DFT methods.^50−52^

**Figure 7:**
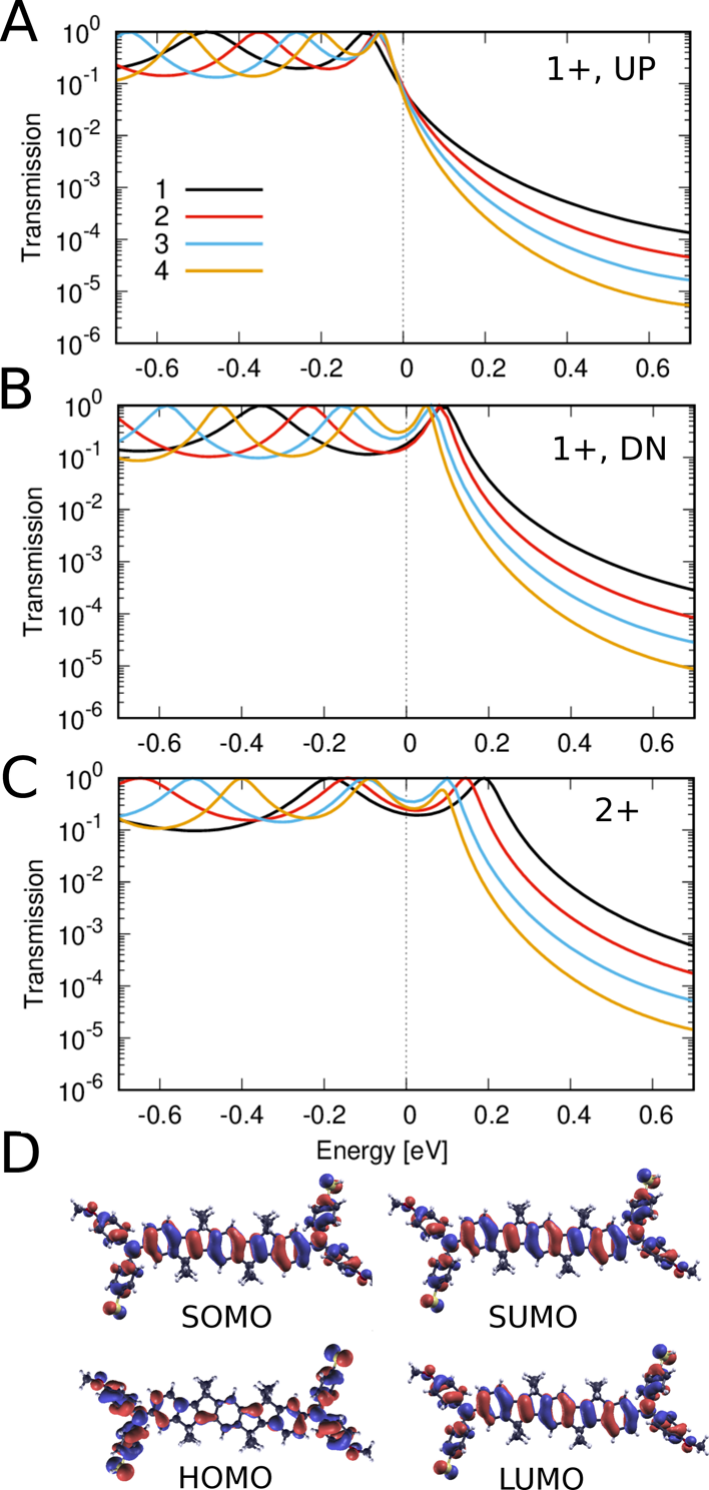
Transmission through singly oxidized SMeDACOPV*n*^+^, where *n* = 1, 2, 3, and 4, for spin down (A) and spin up (B). The total junction conductance is estimated from (*T*(*E*_F_)^UP^ + *T*(*E*_F_)^DN^)/2, and increases with increasing length of the molecule, i.e., going from *n* = 1 to 4. The transmission is dominated by the SOMO and SUMO (D, top) of the two spin components, respectively, which converge in energy with increasing length of the molecule. (C) Spin-degnerate transmission through the doubly oxidized SMeDACOPV*n*^2+^, where *n* = 1, 2, 3, and 4. The conductance *T*(*E*_F_) increases with increasing length of the molecule, i.e., going from *n* = 1 to 3. The transmission is dominated by the HOMO and LUMO (D, bottom) of the molecules, which converge in energy with increasing length of the molecule.

In the case of SMeDACOPV1–4^+^, the presence of spin splitting results in distinct transmission functions for the majority and minority spin components, which we denote as “up” and “down” (Figure 7A,B). We derive the zero-bias conductance of the monocations using . Notably, the frontier resonances are now situated much closer to the Fermi level compared to the neutral forms and can be associated with the singly occupied molecular orbital (SOMO) for spin up and the singly unoccupied molecular orbital (SUMO) for spin down, where we follow the notation of Huo et al.^53^ The SOMO and SUMO represent the split states of the neutral HOMO (Figure 7D). As the length of the molecules increases, the gap between SOMO and SUMO diminishes, bringing both resonances closer to the Fermi level. This shift contributes to an exponential increase in conductance with increasing molecular length.

For SMeDACOPV1–4^2+^, the transmission for both spin up and spin down is degenerate (Figure 7C). The frontier resonances correspond to the HOMO and LUMO, as depicted in (D). Notably, the HOMO–LUMO gap decreases as we progress from *n* = 1 to *n* = 3, and similar to the monocation species, we observe an exponential increase in zero-bias conductance with increasing length. The absolute conductance values and β values for SMeDACOPV1–4^2+^ are slightly higher and lower, respectively, than those of SMeDACOPV1–4^+^ (Figure 3B).

As previously discussed,^33^ the energy gap between the frontier orbitals is attributed to the coupling between the two radical centers of the molecules, which weakens as the molecular length increases. When the coupling diminishes, the frontier orbitals become energetically closer, leading to an enhancement of the transmission at the Fermi level. In the case of SMeDACOPV4^2+^, the coupling is presumed to be so weak that the transmission starts to decline again. This length-dependent behavior of conductance in dicationic one-dimensional TIs has also been explained within the framework of the Su-Schrieffer-Heeger (SSH) model.^54^

## Conclusions

We show that rigidification of the π-system representing the conductance path, consequentially eliminating rotational attenuation of the orbital overlap and therefore enhancing conjugation, leads to a decrease of the overall length dependence in oligophenylene-vinylene molecular wires. This is indicated by a small β parameter (0.05 Å^–1^) of the neutral forms compared to the literature value of 0.17 Å^–1^ for nonrigidified analogs in the same length range.^12^ The oxidized forms show an inverted conductance decay, however only slightly, as the β parameter of −0.02 to −0.03 Å^–1^ is less negative than what is reported for oxidized oligo-phenylenes (−0.21 Å^–1^ for monocations, and −0.07 Å^–1^ for dications).^33^ Furthermore, the oxidized forms exhibit a conductance increase by a factor of up to 1800 with respect to the neutral compounds and excellent conductance values (0.9–2.5 × 10^–2^ G_0_) considering their length of up to 3.6 nm. The trend of inverted conductance decay is broken when increasing the number of repeating units from 3 to 4 due to diminished coupling/redox center communication with increasing distance. The maximum radical distance for inverted conductance decay facilitated by one or two unpaired spins was however increased significantly from 1.4 nm in polyphenylenes to 2.4 nm.^33^ Our results highlight the suitability of oxidized and neutral diarylamine-capped COPVs as a molecular wire by acting as formidable conductors over a long distance, accompanied by excellent signal-to-noise ratios.

## Supporting Information Available

Experimental procedures and characterization data for all new compounds as well as additional analytical data (PDF)
