# Supplementary material for: High Molecular Conductance and Inverted Conductance Decay over 3 nm in Aminium-Terminated Carbon-Bridged Oligophenylene-Vinylenes
Source: J Am Chem Soc. 2024 Dec 20;147(1):957–64. doi: 10.1021/jacs.4c13901 (PMC11726558; doi:10.1021/jacs.4c13901)
Supplement: Supplementary file 1 — ja4c13901_si_001.pdf [file ja4c13901_si_001.pdf]

Supporting Information: High Molecular Conductance and Inverted Conductance Decay over 3 nm in Aminium-Terminated Carbon-Bridged Oligophenylene-Vinylenes

Luisa K. I. Rieger,[†] Susanne Leitherer,[‡] William Bro-Jørgensen,[‡] Gemma C. Solomon,[*,‡,¶] and Rainer F. Winter[*,†]

†*Department of Chemistry, University of Konstanz, 78434 Konstanz, Germany*

‡*Nano-Science Center and Department of Chemistry, University of Copenhagen, DK-2100 Copenhagen, Denmark.*

¶*NNF Quantum Computing Programme, Niels Bohr Institute, University of Copenhagen, DK-2100 Copenhagen, Denmark.*

E-mail: gsolomon@chem.ku.dk; rainer.winter@uni-konstanz.de

Contents

1 Synthetic procedures **S-3**

2 NMR spectra and MALDI-TOF MS **S-10**

3 UV-Vis/NIR spectroscopy **S-15**

4 Cyclic voltammetry **S-16**

5 STM-BJ data **S-17**

6 Computational results **S-22**

References **S-30**

1 Synthetic procedures

All synthetic procedures described in the following were carried out under inert gas using standard Schlenk techniques. Solvents were dried and saturated with inert gas using a MBraun SPS 5/7 solvent purification system (DCM, toluene) or by distillation (MeOH, CCl_4). Starting materials were obtained from commercial suppliers and employed without further purification. Diarylamine-bridged COPVs featuring one to four repeating units (SMeDACOPV1-4) and their precursors were synthesized as reported in the literature[S1–S7] with minor modifications, which are detailed in the following procedures. An overview over all synthetic steps is given in the Schemes S1 and S2. The bromination reagent $CuBr_2$@Al_2O_3 was prepared according to the literature.[S8]

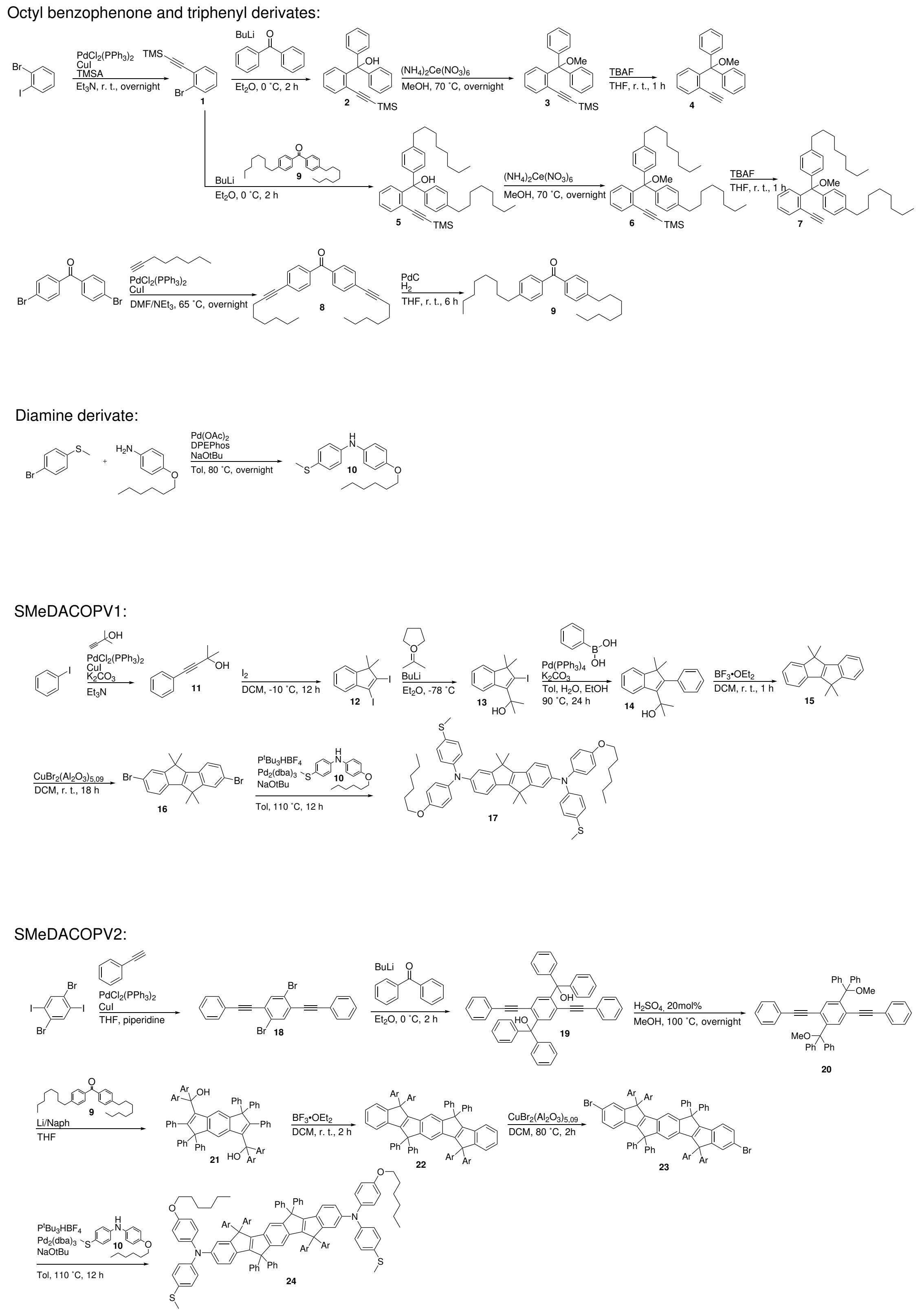

Scheme S1: Schematic overview of synthesis steps required for the triphenyl-, benzophenone- and diarylamine intermediates as well as SMeDACOPV1-2.

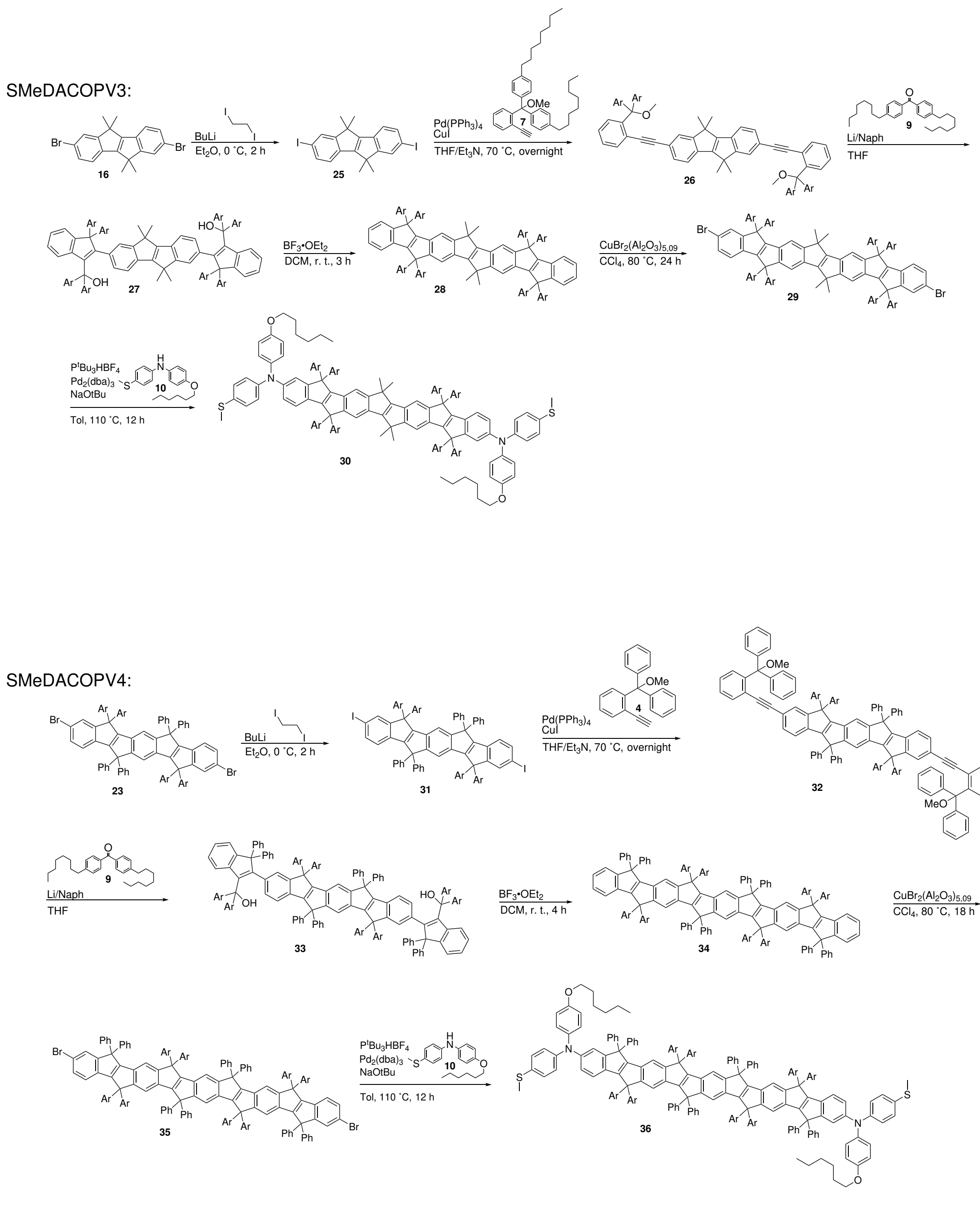

Scheme S2: Schematic overview of synthesis steps required for SMeDACOPV3-4.

1.1 Bromination of COPVs

$CuBr_2$@Al_2O_3 (6.0 mmol) was suspended in a solution of COPVn (1.0 mmol) in the respective solvent (10 mL) and stirred under the respective conditions. The reaction was worked up

according to one of the two procedures below.

In the case of COPV3, five more equivalents of the brominating agent had to be added followed by stirring under the given reaction conditions for 12 h several times to drive the reaction towards completion.

Table S1: Conditions for different COPVn.

SMeDACOPVn	solvent	temperature	reaction time	yield
1	DCM	r. t.	18 h	99 %
2	DCM	80 °C	2 h	95 %
3	CCl_4	90 °C	18 h	62 %
4	CCl_4	80 °C	16 h	79 %

1.1.1 Work up procedure for Br_2COPV1–2:

The mixture was passed through a silica plug with DCM as the solvent and the filtrate was concentrated under reduced pressure to give the title compound as an off-white solid.

1.1.2 Work up procedure for Br_2COPV3–4:

The mixture was passed through a plug of deactivated alumina using a solvent gradient of hexane to DCM and the filtrate was concentrated under reduced pressure to give the respective title compound as a yellow solid.

1.2 Synthesis of ((2,5-bis(phenylethynyl)-1,4-phenylene)bis-(methoxymethanetriyl))tetrabenzene (20)

A suspension of compound **19** (2.82 g, 4.4 mmol, 1.00 eq) in 20 mol% H_2SO_4 (0.05 mL, 0.09 g, 0.2 eq) in $MeOH_{abs}$ (80 mL) was refluxed under inert gas atmosphere overnight. After cooling to room temperature, DCM was added until all solid had dissolved. The resulting mixture was extracted with saturated $NaHCO_3$ solution (3 x 80 mL), dried over $MgSO_4$ and concentrated under reduced pressure. The residue was purified via recrystallization from ethyl acetate to afford 20 as a white crystalline solid in 85 % yield.

1.3 Synthesis of SMeDACOPVn

Br_2COPVn (0.016 mmol, 1.0 eq), diarylamine **10** (0.042 mmol, 2.5 eq, Scheme S1, top middle), Pd_2dba_3 (0.05 eq), $^tBu_3PHBF_4$ (0.001 mmol) and NaO^tBu (0.042 mmol) were suspended in degassed toluene and stirred at 110 °C for 18 h under inert gas atmosphere. The reaction mixture was subjected to the respective work up procedure as specified below.

1.3.1 Work up procedure for SMeDACOPV1 (17):

The solvent was evaporated under reduced pressure and the crude product was filtered through a silica plug with hexane/DCM 1:1. After solvent evaporation, the crude product was washed with hot MeCN and recrystallized from EtOH/ethyl acetate to afford SMeDACOPV1 as a pale yellow solid in a yield of 45 %.

^{1}H-NMR (600 MHz, CD_2Cl_2) δ (ppm) = 7.17 (d, J = 8.1 Hz, 2H), 7.16 – 7.13 (m, 6H), 7.06 (d, J = 8.9 Hz, 4H), 6.96 (d, J = 8.7 Hz, 4H), 6.87 (dd, J = 8.0, 2.1 Hz, 2H), 6.84 (d, J = 8.9 Hz, 4H), 3.94 (t, J = 6.6 Hz, 4H), 2.45 (s, 6H), 1.81 – 1.73 (m, 4H), 1.49 – 1.44 (m, 4H), 1.42 (s, 12H), 1.37 – 1.33 (m, 8H), 0.93 – 0.90 (m, 6H).

^{13}C-NMR (151 MHz, CD_2Cl_2) δ (ppm) = 160.56, 156.20, 154.64, 146.96, 145.55, 140.97, 133.13, 130.13, 129.05, 127.29, 123.07, 122.29, 119.49, 118.38, 115.65, 68.70, 45.18, 32.02, 29.72, 26.11, 24.65, 23.01, 17.36, 14.20.

MALDI-TOF MS Calcd. for [M^+]: 886.5, found: 886.4

1.3.2 Work up procedure for SMeDACOPV2 (24):

The solvent was evaporated under reduced pressure and the crude product was subjected to column chromatography over silica with hexanes/DCM 3:1. The obtained solid was washed with hot MeCN to afford SMeDACOPV2 as a bright yellow solid in a yield of 50 %.

^{1}H-NMR (800 MHz, C_6D_6) δ (ppm) = 7.73 (d, J = 2.1 Hz, 2H), 7.72 (s, 2H), 7.43 (d, J = 8.2 Hz, 8H), 7.40 (d, J = 6.9 Hz, 8H), 7.29 (d, J = 8.4 Hz, 2H), 7.05 (d, J = 8.7 Hz, 4H), 6.99 – 6.92 (m, 28H), 6.91 (dd, J = 8.4, 2.1 Hz, 2H), 6.72 (d, J = 8.9 Hz, 4H), 3.61 (t, J = 6.4 Hz, 4H), 2.46 (t, J = 7.9 Hz, 8H), 2.02 (s, 6H), 1.62 – 1.57 (m, 4H), 1.53 – 1.47 (m, 8H), 1.34 – 1.27 (m, 12H), 1.26 – 1.16 (m, 40H), 0.91 (t, J = 7.3 Hz, 12H), 0.87 (t, J = 7.2 Hz, 6H).

^{13}C-NMR (201 MHz, C_6D_6) δ (ppm) = 159.32, 156.68, 156.36, 155.06, 154.79, 146.70, 146.48, 144.42, 141.61, 141.38, 140.89, 137.23, 133.64, 131.38, 129.23, 129.15, 128.82, 128.69, 128.35, 128.29, 128.11, 127.99, 127.45, 126.95, 124.16, 121.87, 121.60, 120.95, 118.42, 115.66, 68.11, 63.74, 63.21, 36.05, 32.35, 31.93, 31.79, 29.94, 29.89, 29.67, 29.65, 26.14, 23.13, 23.00, 16.81, 14.42, 14.27.

MALDI-TOF MS Calcd. for [M^+]: 2013.2, found: 2013.5

1.3.3 Work up procedure for SMeDACOPV3 (30):

The solvent was evaporated under reduced pressure and the crude product was subjected to column chromatography over deactivated alumina using a solvent gradient from hexanes to hexanes/DCM 10:1 to afford SMeDACOPV3 as a deep yellow solid in a yield of 48 %.

^{1}H-NMR (600 MHz, C_6D_6) δ (ppm) = 7.72 (s, 2H), 7.64 (d, J = 2.1 Hz, 2H), 7.58 (s, 2H), 7.54 (d, J = 7.2 Hz, 16H), 7.28 (d, J = 8.4 Hz, 2H), 7.02 - 7.00 (m, 12H), 6.87 - 6.83 (m, 16H), 6.82 (dd, J = 8.3, 2.1 Hz, 2H), 6.66 (d, J = 9.0 Hz, 4H), 3.58 (t, J = 6.4 Hz, 4H), 2.47 (t, J = 7.8 Hz, 8H), 2.33 (t, J = 7.8 Hz, 8H), 1.99 (s, 6H), 1.59 – 1.48 (m, 12H), 1.43 (s, 12H), 1.38 - 1.33 (m, 8H), 1.31 – 1.09 (m, 92H), 0.87 (m, 24H), 0.83 (t, J = 7.2 Hz, 6H).

^{13}C-NMR (151 MHz, C_6D_6) δ (ppm) = 159.34, 158.44, 157.32, 155.92, 155.15, 154.63, 146.17, 141.29, 141.20, 141.04, 140.56, 136.58, 136.08, 133.36, 130.81, 128.89, 128.79, 128.46,

128.44, 128.10, 127.25, 127.20, 127.05, 123.71, 121.55, 121.13, 120.48, 116.25, 115.25, 114.54, 67.74, 62.99, 62.88, 44.68, 35.68, 35.57, 31.95, 31.91, 31.57, 31.47, 31.22, 29.54, 29.51, 29.47, 29.32, 29.25, 25.77, 24.49, 22.75, 22.73, 22.63, 16.51, 14.03, 13.90.

MALDI-TOF MS Calcd. for [M^+]: 2643.8, found: 2643.5

1.3.4 Work up procedure for SMeDACOPV4 (36):

The solvent was evaporated under reduced pressure and the crude product was subjected to column chromatography over deactivated alumina using a solvent gradient from hexanes/DCM 10:1 to hexanes/DCM 2:1 to afford SMeDACOPV4 as a bright orange solid in a yield of 48 %.

^{1}H-NMR (600 MHz, C_6D_6) δ (ppm) = 7.84 (s, 2H), 7.80 (s, 2H), 7.78 (s, 2H), 7.67 (d, 2H, J = 2.2 Hz), 7.47 - 7.44 (m, 8H), 7.39 - 7.37 (m, 18H), 7.33 (d, 8H, J = 8.2 Hz), 7.12 - 7.10 (m, 12H), 7.04 (d, 4H, J = 8.7 Hz), 7.02 - 7.68 (m, 14H), 6.92 (d, 4H, J = 6.8 Hz), 6.90 (d, 4H, J = 6.4 Hz), 6.85 (d, 8H, J = 8.1 Hz), 6.81 (d, 8H, J = 8.3 Hz), 6.71 (d, 4H, J = 9.0 Hz), 3.60 (t, 4H, J = 6.4 Hz), 2.40 (t, 8H, J = 7.7 Hz), 2.34 (t, 8H, J = 7.2 Hz), 2.00 (s, 6H), 1.61 - 1.55 (m, 4H), 1.44 - 1.10 (m, 108H), 0.95 - 0.89 (m, 24H), 0.86 (t, 6H, J = 7.23).

^{13}C-NMR (151 MHz, C_6D_6) δ (ppm) = 158.87, 157.25, 157.05, 156.53, 156.38, 155.91, 155.75, 155.22, 154.28, 146.76, 146.46, 144.65, 144.34, 141.75, 141.53, 141.51, 141.45, 140.89, 137.49, 137.15, 137.13, 133.87, 131.45, 129.44, 129.34, 129.26, 129.17, 129.13, 128.82, 128.77, 128.71, 128.66, 128.47, 127.61, 127.57, 127.45, 127.10, 126.93, 124.18, 122.03, 121.83, 120.88, 118.70, 118.57, 118.30, 115.68, 77.66, 77.44, 77.23, 68.11, 63.80, 63.74, 63.21, 63.12, 36.00, 35.93, 32.38, 32.34, 31.97, 31.91, 31.67, 31.64, 29.93, 29.89, 29.74, 29.66, 29.63, 26.12, 23.16, 23.13, 23.06, 22.99, 16.79, 14.44, 14.41, 14.36, 14.26.

MALDI-TOF MS Calcd. for [M$^+$]: 3322.0, found: 3322.5

2 NMR spectra and MALDI-TOF MS

NMR spectra were recorded on BRUKER AVANCE III 400 MHz and BRUKER AVANCE III 600 MHz spectrometers at 298 K. Residual proton signals of the employed deuterated solvent serve as the reference. Spectral data processing was carried out using the Mnova software (Mestrelab Research).

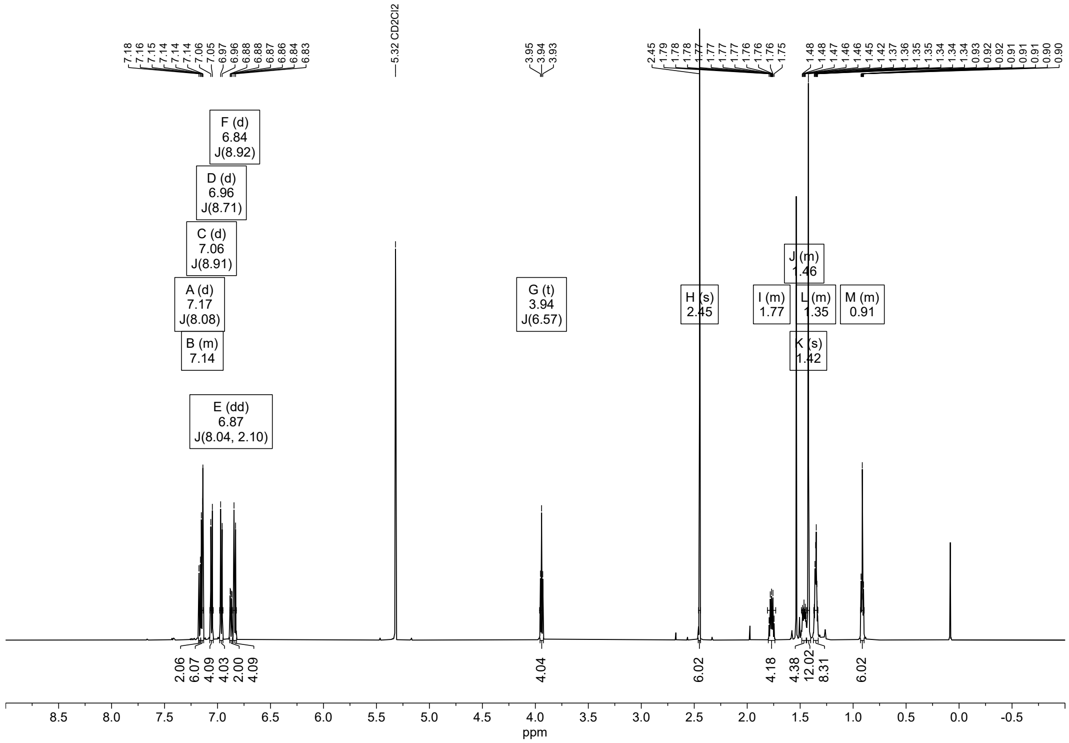

Figure S1: ^{1}H-NMR spectrum (600 MHz, 298 K, CD_2Cl_2) of SMeDACOPV1 (17).

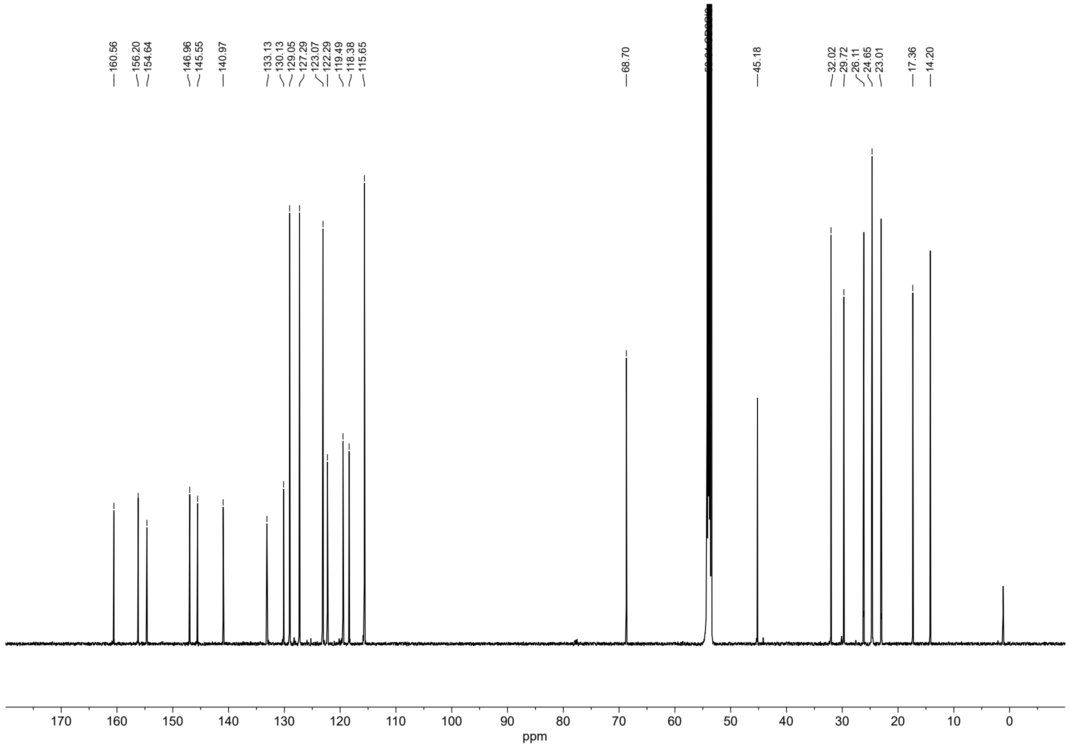

Figure S2: ^{13}C-NMR spectrum (151 MHz, 298 K, CD_2Cl_2) of SMeDACOPV1 (17).

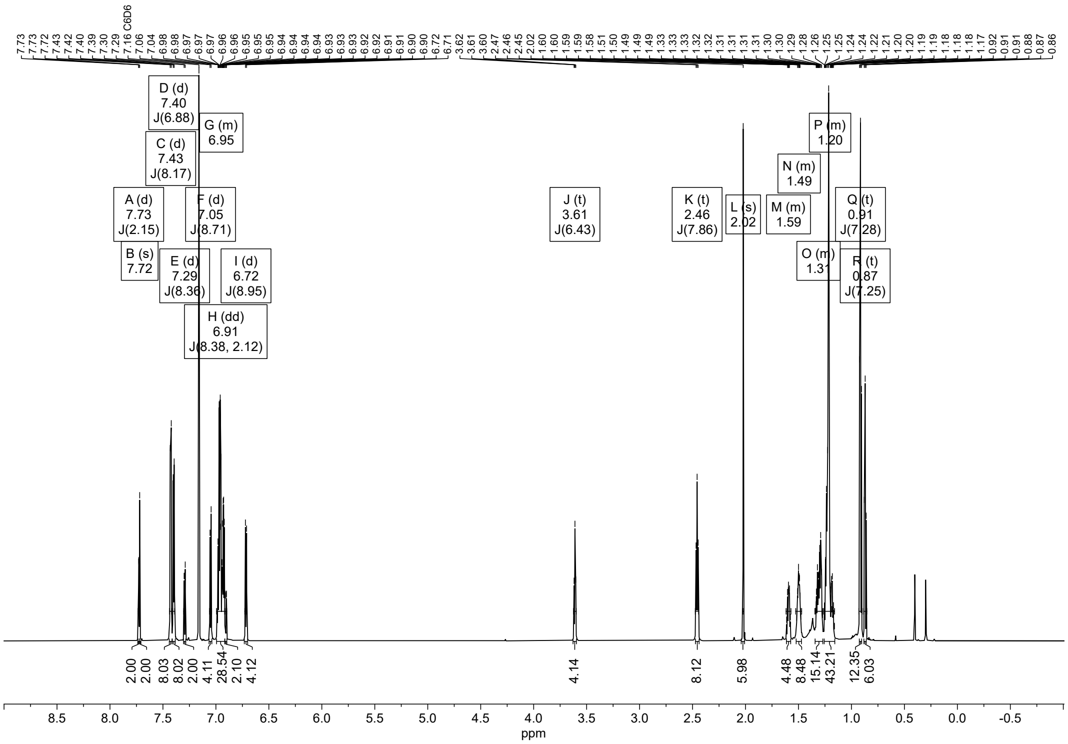

Figure S3: ^{1}H-NMR spectrum (800 MHz, 298 K, C_6D_6) of SMeDACOPV2 (24).

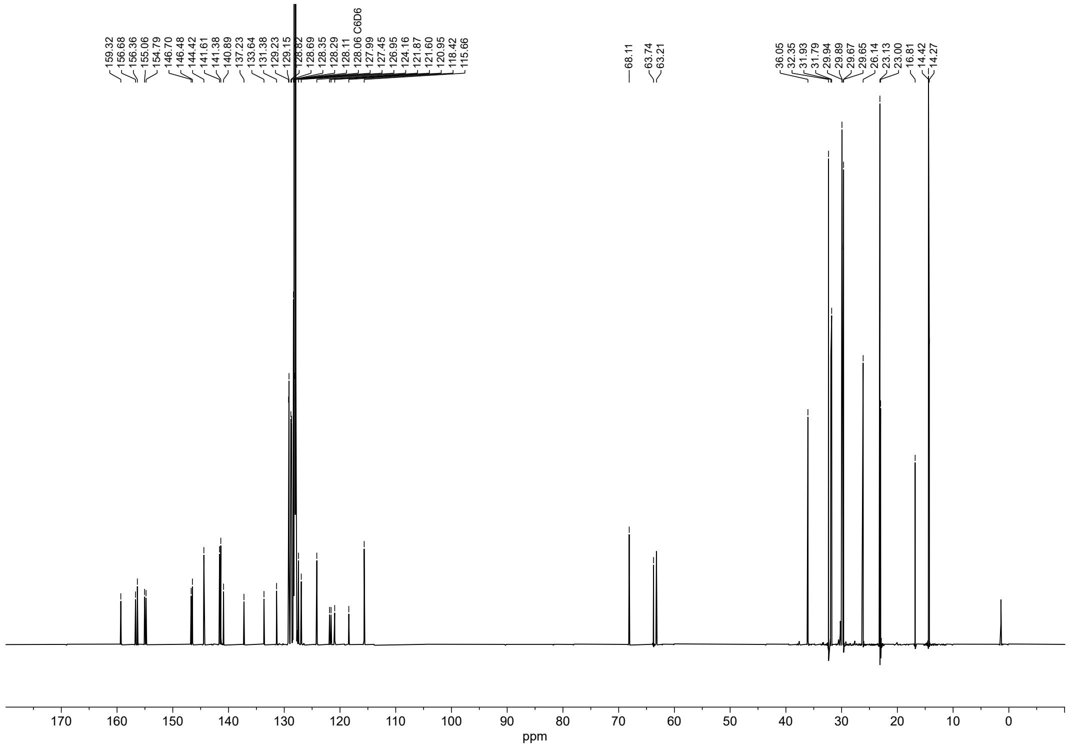

Figure S4: ^{13}C-NMR spectrum (201 MHz, 298 K, C_6D_6) of SMeDACOPV2 (24).

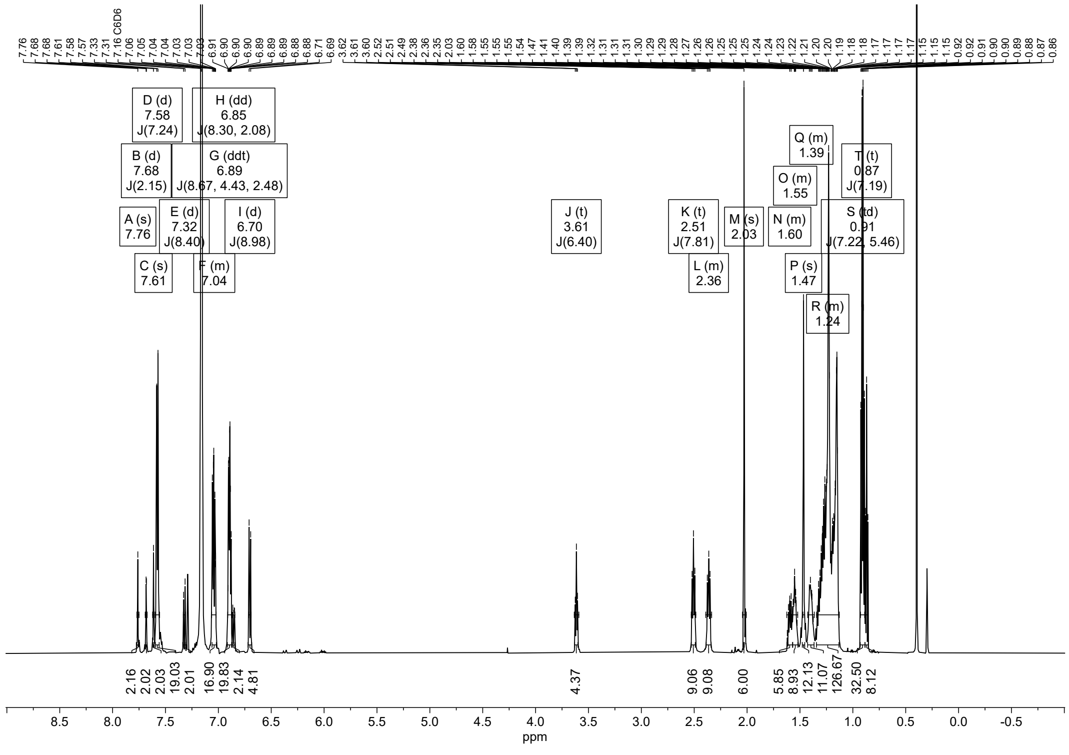

Figure S5: ^{1}H-NMR spectrum (600 MHz, 298 K, C_6D_6) of SMeDACOPV3 (30).

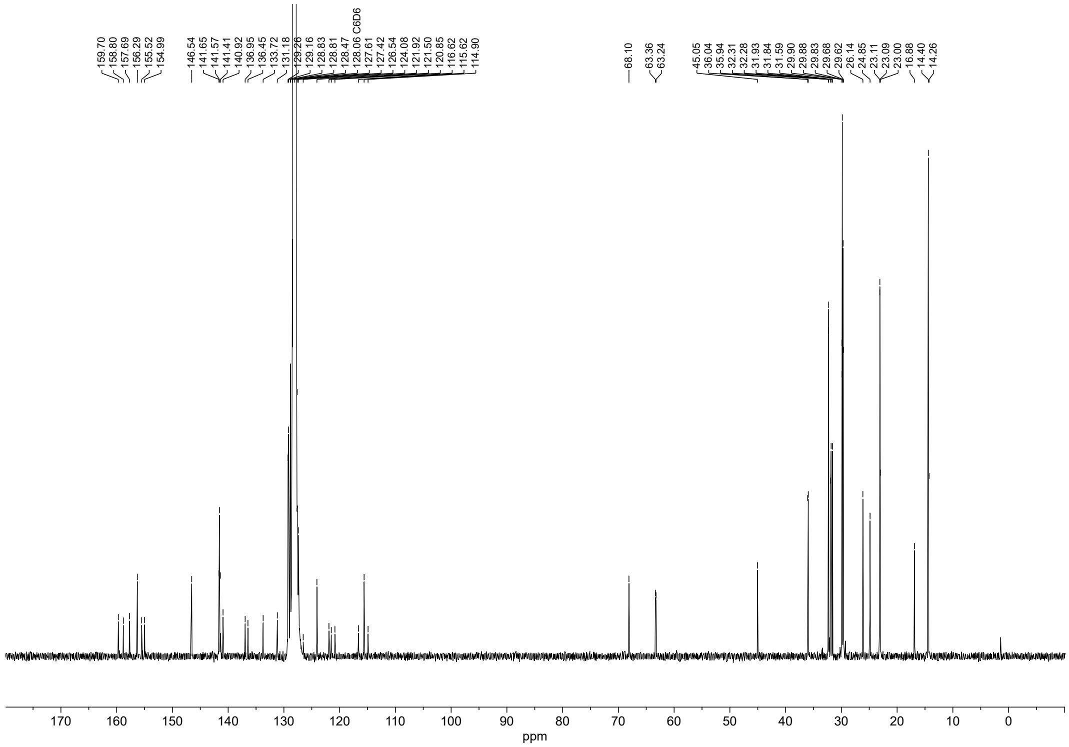

Figure S6: ^{13}C-NMR spectrum (151 MHz, 298 K, C_6D_6) of SMeDACOPV3 (30).

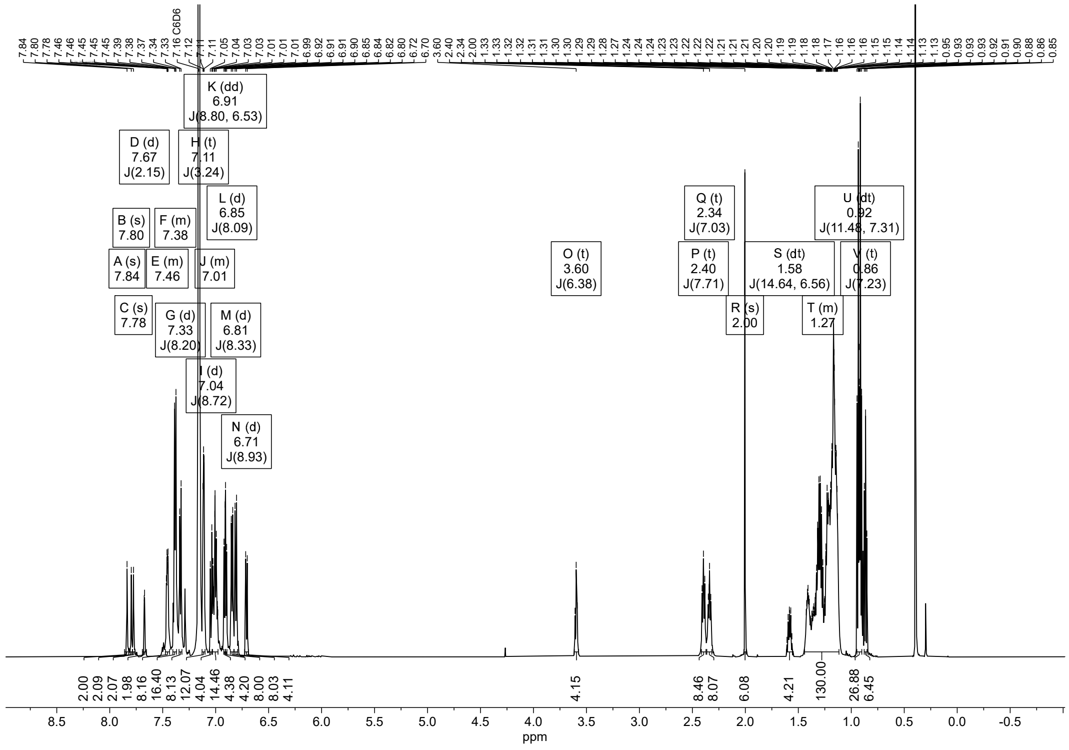

Figure S7: ^{1}H-NMR spectrum (600 MHz, 298 K, C_6D_6) of SMeDACOPV4 (36).

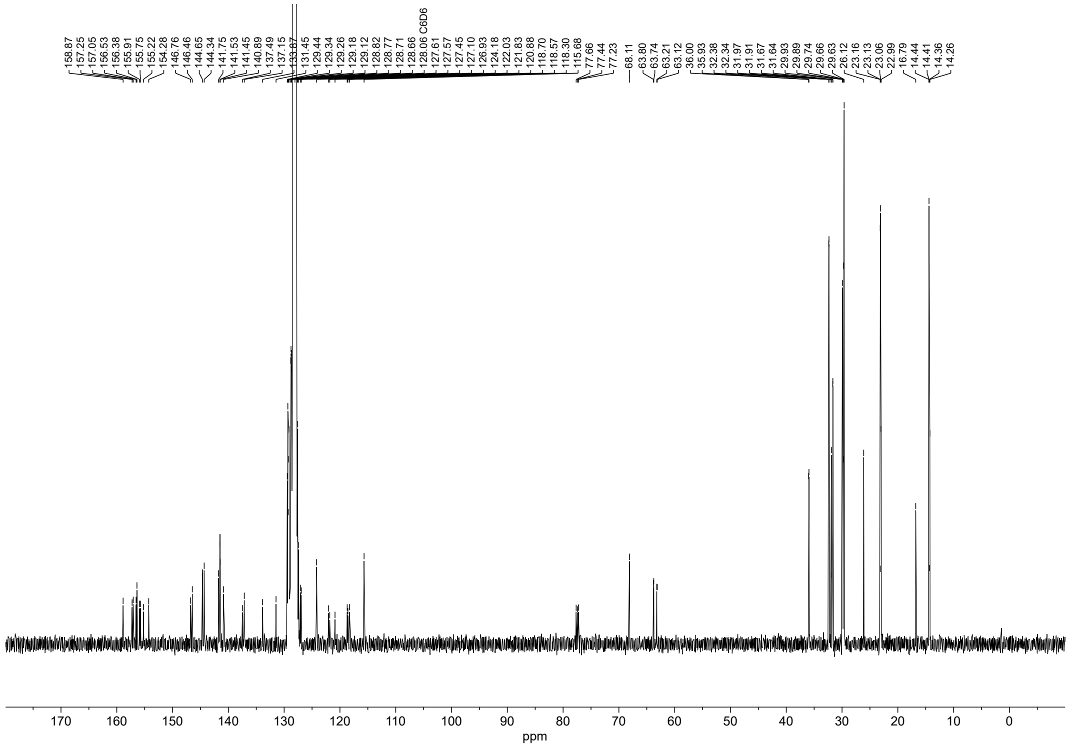

Figure S8: ^{13}C-NMR spectrum (151 MHz, 298 K, C_6D_6) of SMeDACOPV4 (36).

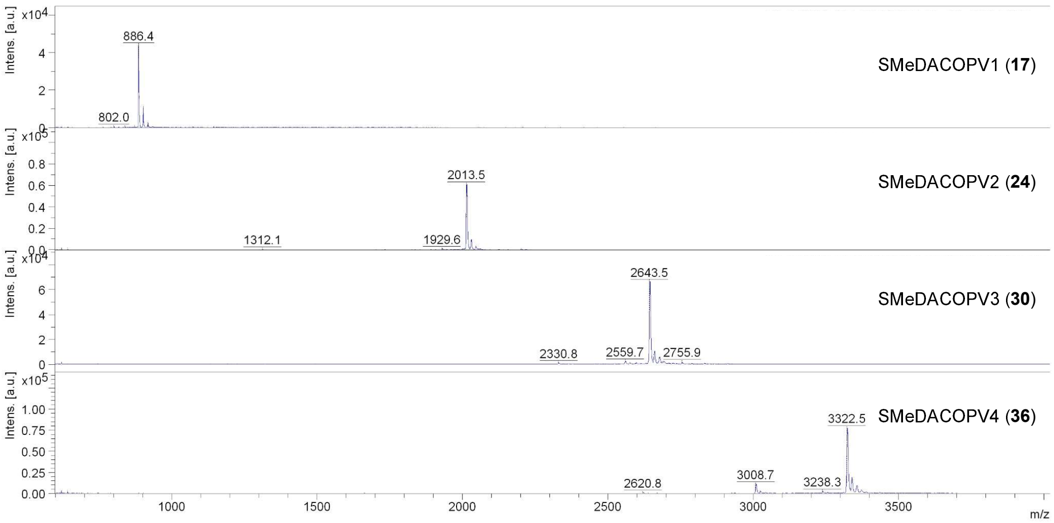

Figure S9: MALDI-TOF mass spectra of SMeDACOPV1-4 measured from a α-cyano-4-hydroxycinnamic acid matrix in positive-ion-mode.

3 UV-Vis/NIR spectroscopy

UV-Vis/NIR spectra were obtained using a TIDAS fiber optic diode array spectrometer, comprising a combination of MCS UV/NIR and PGS NIR instruments from J&M. The NIR region from 2000 to 3500 nm was measured using a Bruker Tensor II FT-IR spectrometer. The two datasets were normalized and merged to a single curve at the 2000 nm datapoint for SMeDACOPV2/3 and at 1800 nm for SMeDACOPV4. DCM was used as the solvent unless stated otherwise.

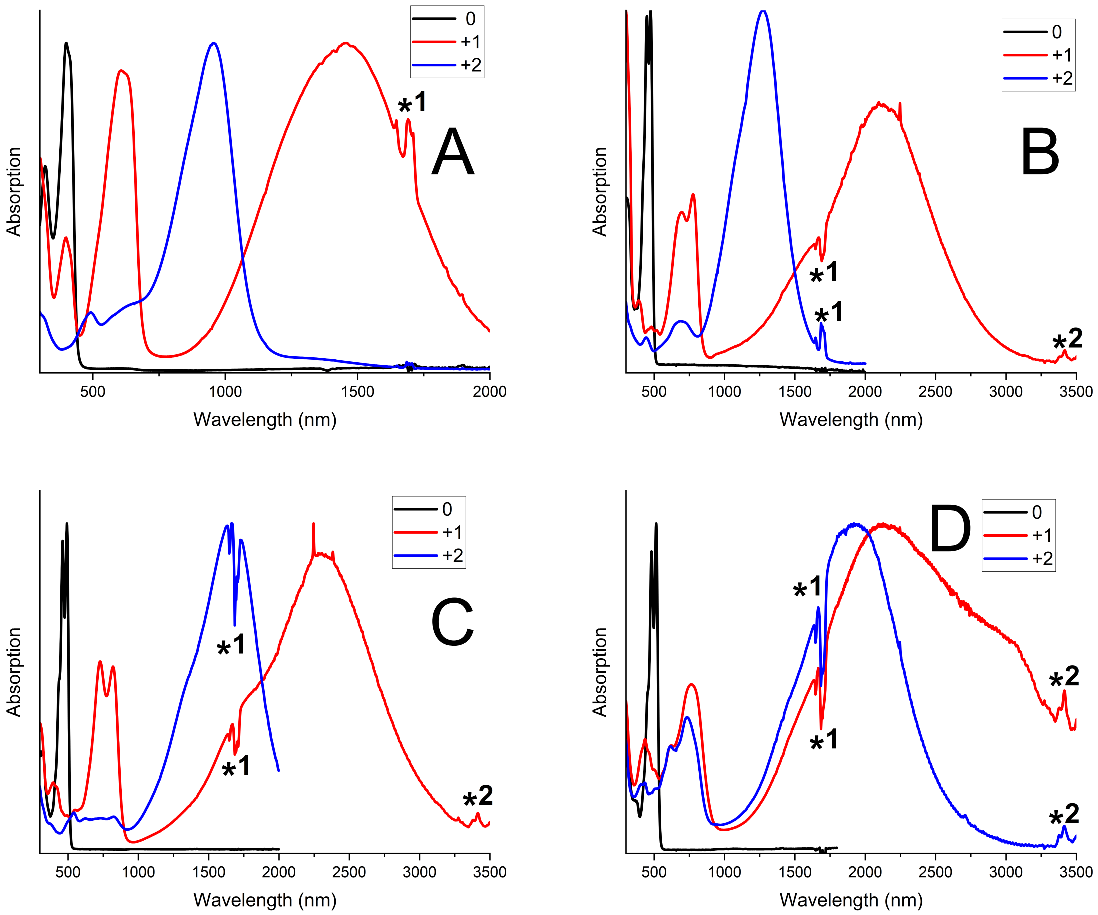

Figure S10: Normalized UV-Vis/NIR spectra of the neutral, singly and doubly oxidized forms of SMeDACOPV1 (A), SMeDACOPV2 (B), SMeDACOPV3 (C), and SMeDACOPV4 (D). *1 denotes a set-up artefact and *2 is caused by ambient CO_2.

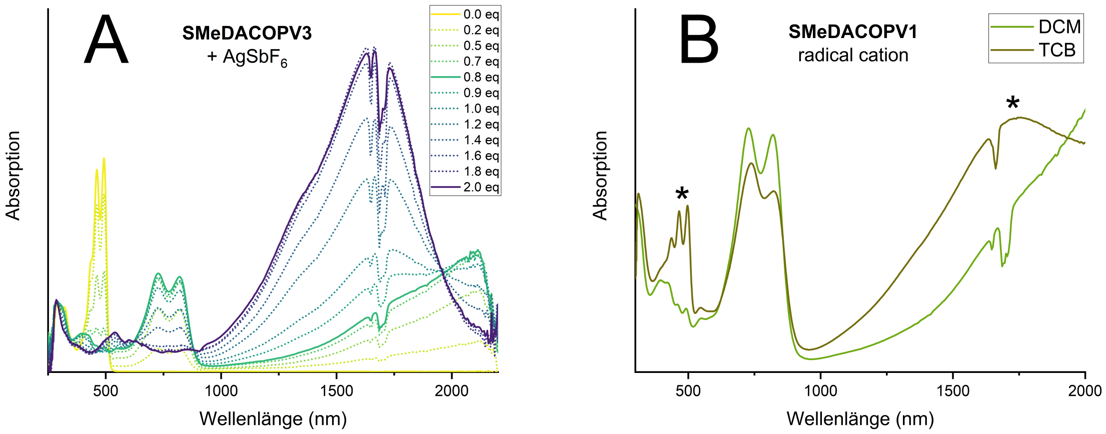

Figure S11: (A) Exemplary preparatory titration curve of SMeDACOPV3. Samples were removed after each addition of $AgSbF_6$ and a spectrum was recorded. Spectra are normalized to the maximum at 285 nm, where all species absorb in equal intensity as seen in the literature.[S2] (B) Comparison of UV-Vis/NIR spectra of the radical cation of SMeDACOPV1 in DCM and TCB. The spectrum recorded in TCB indicates partial disproportionation, as seen from the presence of features associated with the neutral and the dicationic form (marked with an asterisk).

Spectra correspond to what is reported in the literature[S2] of molecules whith -SMe instead of -OHex at the N-bonded phenyl ring (see Figure S10).

4 Cyclic voltammetry

Cyclic voltammetry was performed under inert gas atmosphere. Glassy carbon, platinum and Ag/AgCl served as working, counter and reference electrode materials, respectively. $TBAPF_6$ served as electrolyte in dichloromethane as the solvent (0.1 M). Oxygen was removed by bubbling argon via a cannula for 2 min prior to measurement. Decamethylferrocene was used as internal standard for referencing. All potentials are converted to the ferrocene/ferrocinium potential scale with consideration of the half-wave potential of -0.540 V for $Cp^*{}_2Fe^{0/+}$ vs. $Cp_2Fe^{0/+}$.

Table S2: Oxidation potentials for the amine- and bridge-centered oxidations of DACOPVn against the ferrocene/ferrocinium redox pair in DCM using 0.1 M $TBAPF_6$ as electrolyte. ΔE denotes the potential difference between the first and the second amine oxidations.

n	1st (mV)	2nd (mV)	3rd (mV)	4th (mV)	5th (mV)	ΔE (mV)
1	-74	139	-	-	-	213
2	36	167	783	1040	-	131
3	-7	116	496	865	-	123
4	57	137	410	728	1095	80

5 STM-BJ data

5.1 General remarks

Molecular conductance measurements were performed according to the STM-BJ method[S9] using a customized set-up[S10] according to the model developed by the group of Latha Venkataraman.[S11–S13]

At the start of each measurement, an electrochemically etched gold tip was prepared by electrolysis of a 2 cm long Au wire (Chempur, 99.9 %, 0.25 mm diameter) submerged into a 1:1 solution of $HCl_{(conc.)}/EtOH_{(abs.)}$ by 1 cm. Application of a voltage of 2.5 V causes a progressing thinning of the submerged wire, eventually resulting in the submerged part to dissolve completely or to rupture from the remaining part at the liquid/gas interface. The resultant tip is rinsed with distilled water and $EtOH_{(abs)}$ three times before use. The Au substrate is cleaned via application of piranha solution (H_2SO_4/H_2O_2 1:1) for 20 min before rinsing with distilled water and $EtOH_{(abs)}$.

The background noise is monitored by measurement of 1000 traces of gold contact only and 1,2,4-trichlorobenzene (TCB) each (Figure S12). Analyte solutions are prepared in concentrations of 1 mM for neutral molecules and 0.01 M for the oxidized forms to suppress background currents while the molecular feature is preserved. For neutral molecules, solutions are ultrasonificated for 1 h with zinc dust and are applied as a suspension to avoid

in situ oxidation by the applied bias voltage that is observed without zinc treatment (see Figure S15).

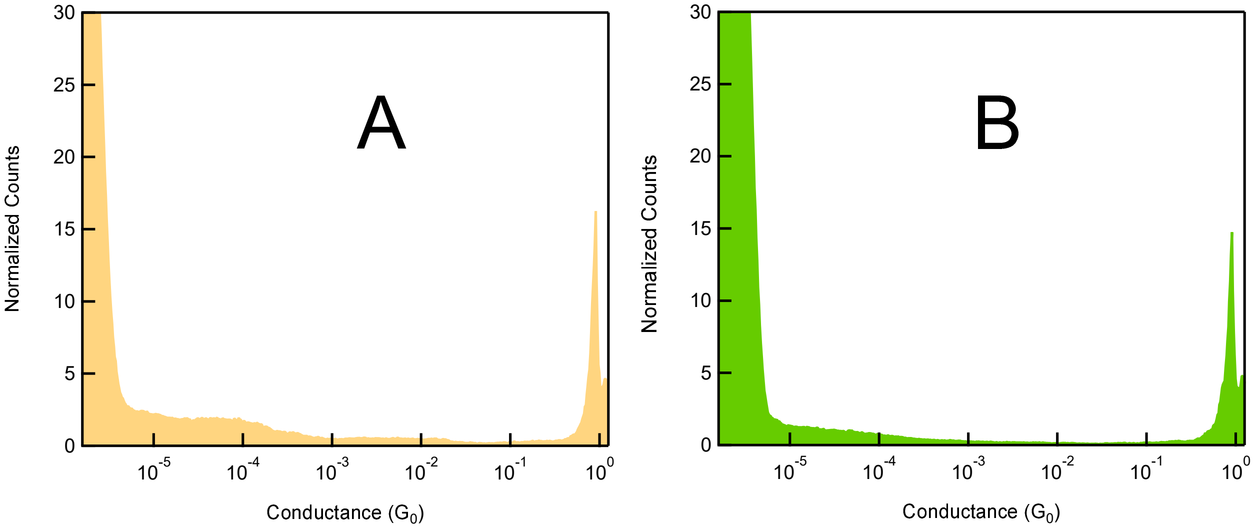

Figure S12: Exemplary histograms depicting the background of the neat gold contact (A) and of 1,2,4-trichlorobenzene (B).

5.2 Neutral tetramer

No molecular features were observed in measurements of neutral SMeDACOPV4 at 100 mV (Figure S13). Measurements at higher bias voltages lead to in situ oxidation. The method of suspending zinc powder in the sample in order to prevent oxidation was found to be ineffective at 250 mV.

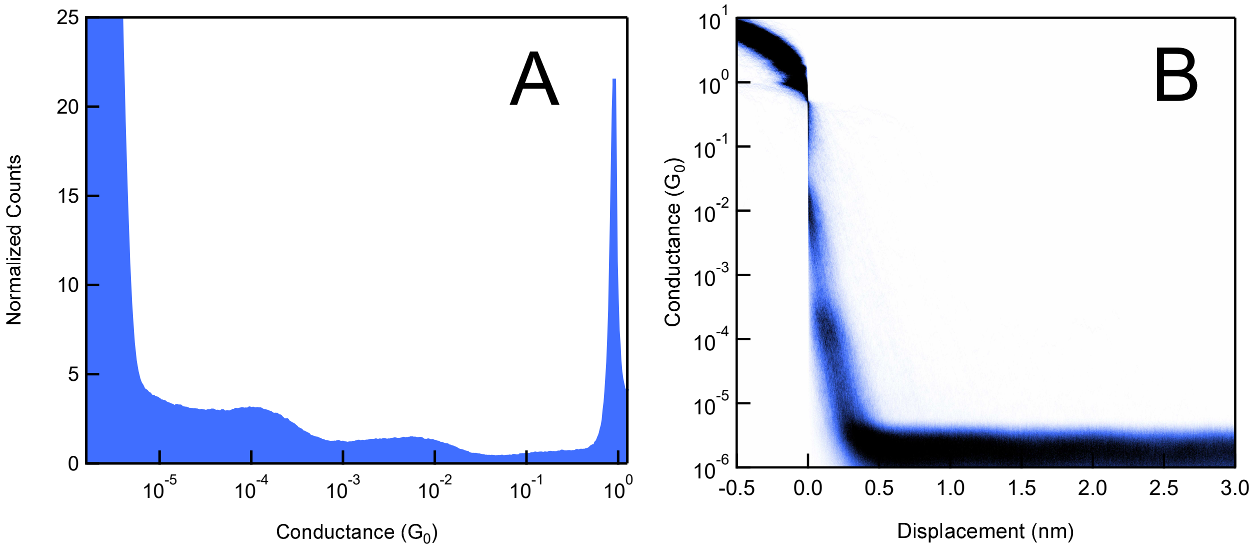

Figure S13: STM-BJ measurement of neutral SMeDACOPV4. No molecular feature is observed.

5.3 Conductance data of radical cations

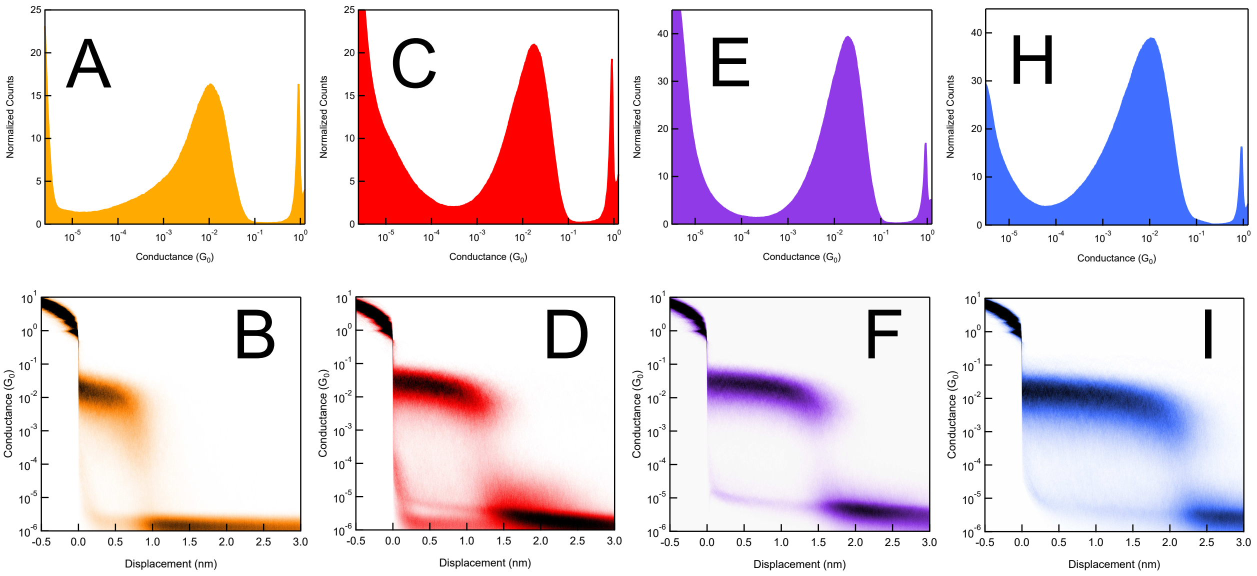

Figure S14: Histograms of STM-BJ measurements on singly oxidized SMeDACOPVn ((A,B): SMeDACOPV1^{1+}, (C,D): SMeDACOPV2^{1+}, (E,F): SMeDACOPV3^{1+}, (G,H): SMeDACOPV4^{1+}) at $V = 100\,\mathrm{mV}$ in TCB.

5.4 Bias variation

In an effort to distinguish the conductance characteristics of the singly and doubly oxidized states, measurements at higher bias voltages were performed for SMeDACOPV3 in order to potentially stabilize the dication. Samples of chemically oxidized compound were found to decompose within the first 100 traces at bias voltage $V = 250\,\mathrm{mV}$ or higher. This was circumvented by in situ oxidation of the neutral compound through the applied bias voltage. This resulted in conductance features that resemble the ones acquired for oxidized samples at $V = 100\,\mathrm{mV}$, with conductance values however appearing at slightly lower values (Figure S15). No additional conductance feature or signal shift to higher conductance values (as predicted for the dication by transport calculations) was observed at increasing bias voltage. A bias voltage of $V = 750\,\mathrm{mV}$ shows a significantly altered profile, indicating decomposition. Changing the solvent to bromonaphthalene (BN) allows for measurements of chemically preperated dicationic SMeCOPV3^{2+} at $V = 500\,\mathrm{mV}$ without decomposition. The observed conductance peak was identical to the value observed at 100 mV bias in BN as well as to the value of (Figure S16)chemically oxidized SMeDACOPV3^{2+} in TCB at 100 mV within experimental uncertainty.

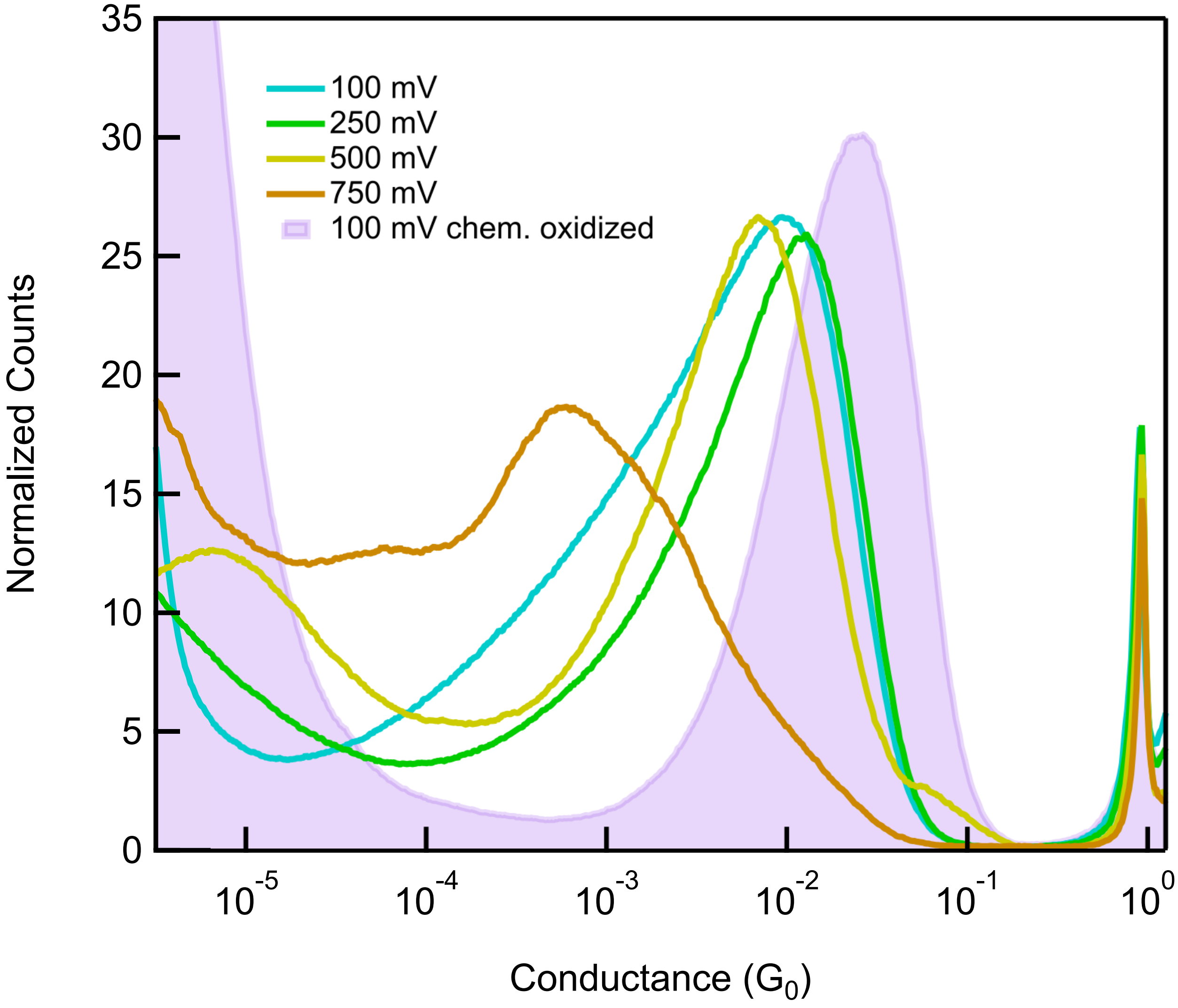

Figure S15: 1D Histograms of SMeDACOPV3 applied in its neutral form and oxidized in situ at different bias voltages in comparison to the chemically oxidized sample at $V = 100\,\text{mV}$. The measurement at $V = 750\,\text{mV}$ indicates significant decomposition.

5.5 Bromonaphthalene as solvent

Measurements of singly and doubly oxidized SMeDACOPV3 in bromonaphthalene reveal a similar but slightly lower conductance values of around 1.7 x 10^{-2} G_0 in comparison to data recorded for solutions in TCB (see Figure S16). The neutral form is almost silent in bromonaphthalene, similar to what is observed for the neutral tetramer in TCB. Measurement of the doubly oxidized form at $V = 500\,\text{mV}$ reveals the same conductance as for 100 mV.

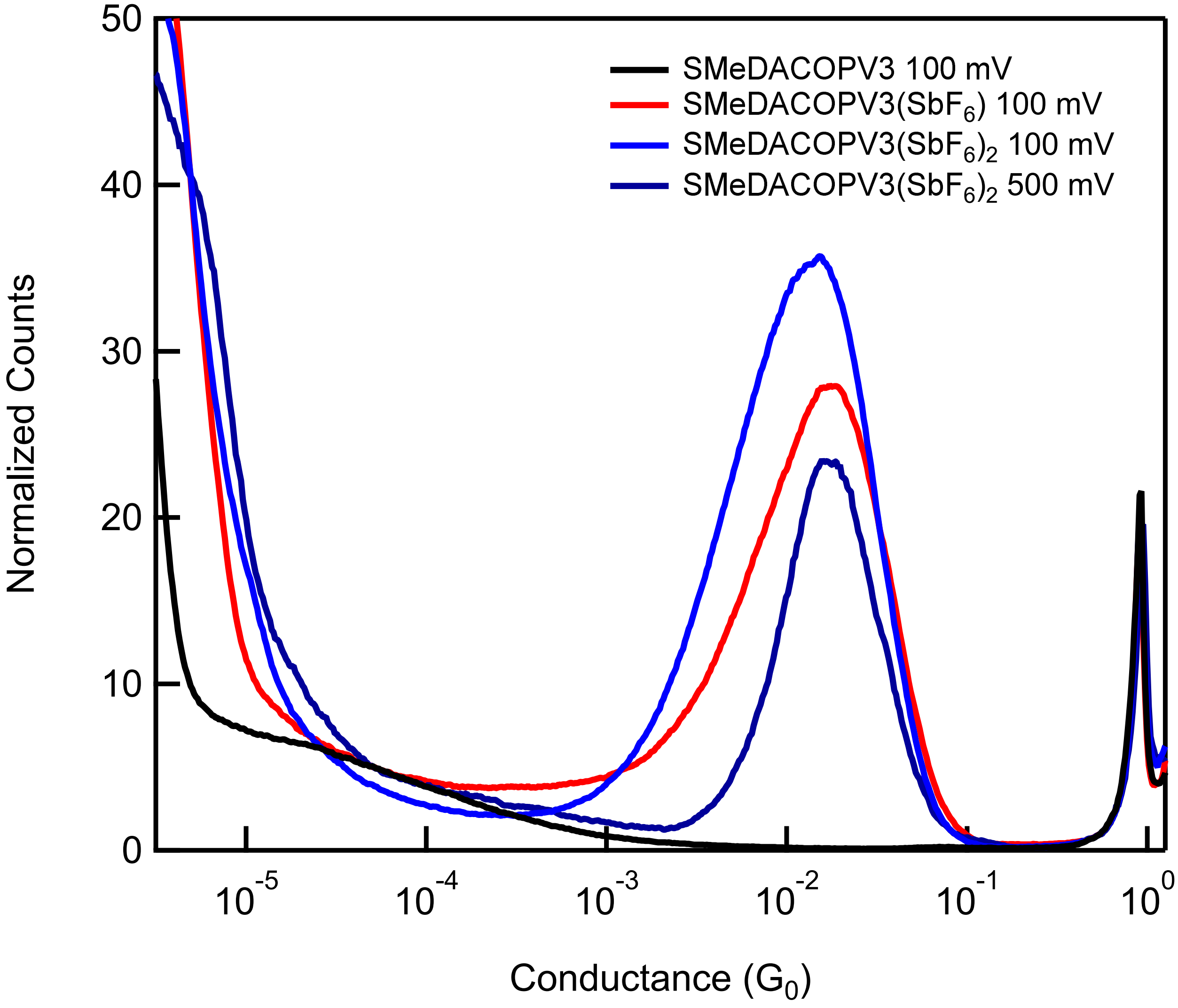

Figure S16: 1D Histograms of neutral, singly and doubly oxidized SMeDACOPV3 measured in bromonaphtaline. The dication was also measured at $V = 500\,\mathrm{mV}$ (2000 traces).

6 Computational results

6.1 Methods

Density functional electronic structure calculations of the molecules in the gas phase were performed using SIESTA,[S14] with the PBE-GGA functional for exchange-correlation and the standard DZP basis-set. The mesh cutoff was set to 300 Ry. To obtain the oxidized species, the net charge was increased by +1/+2 and spin polarization was enabled. The geometries

of the isolated molecules were relaxed in vacuum with a force tolerance of 0.01 V/Å. Post-processing analysis was done using the SISL[S15] code to obtain the Hamiltonian H and overlap matrix S for the transport calculations. The transmission was calculated using

$$T(E) = Tr\{\Gamma_{\mathrm{R}}\mathrm{G(E)}\Gamma_{\mathrm{L}}\mathrm{G(E)}^{\dagger}\}, \tag{1}$$

where the Green's function is defined as

$$\mathrm{G(E)} = [S \cdot E - H - \Sigma_L - \Sigma_R]^{-1} \tag{2}$$

We describe the electrode-molecule interaction in the wide-band-approximation, which is a good approximation for gold electrodes. In particular, the matrix elements of the self-energies $\Sigma_\alpha, \alpha = L, R$ in a local basis (represented by atomic orbitals $|\chi\rangle_\nu$) are given by

$$(\Sigma_\alpha)_{\nu\nu} = -\frac{i}{2}(\Gamma_\alpha)_{\nu\nu}, \tag{3}$$

with $(\Gamma_\alpha)_{\nu\nu} = 1$ eV for orbitals ν corresponding to the outermost S atom on the left and right side of the molecule, respectively.

We utilize the Fermi energy calculated by SIESTA, which is positioned centrally within the gap between the highest occupied and the lowest unoccupied state, and is influenced by factors such as electronic temperature and electronic occupancies.[S16] It is important to note that the conclusions drawn from our results remain broadly valid across a considerable energy range between the molecular frontier orbitals.

The geometries are publicly available at https://iochembd.chem.ku.dk/browse/handle/100/197 and the script for the WBA transport calculations can be found at https://github.com/Solomon-Hansen/WBA-Trans.

6.2 Molecular orbitals

Fig. S17 displays the molecular frontier orbitals (HOMO,LUMO/SOMO,SUMO) and their energy gap, of isolated SMeDACOPV1-4 in the neutral, singly and doubly oxidized form.

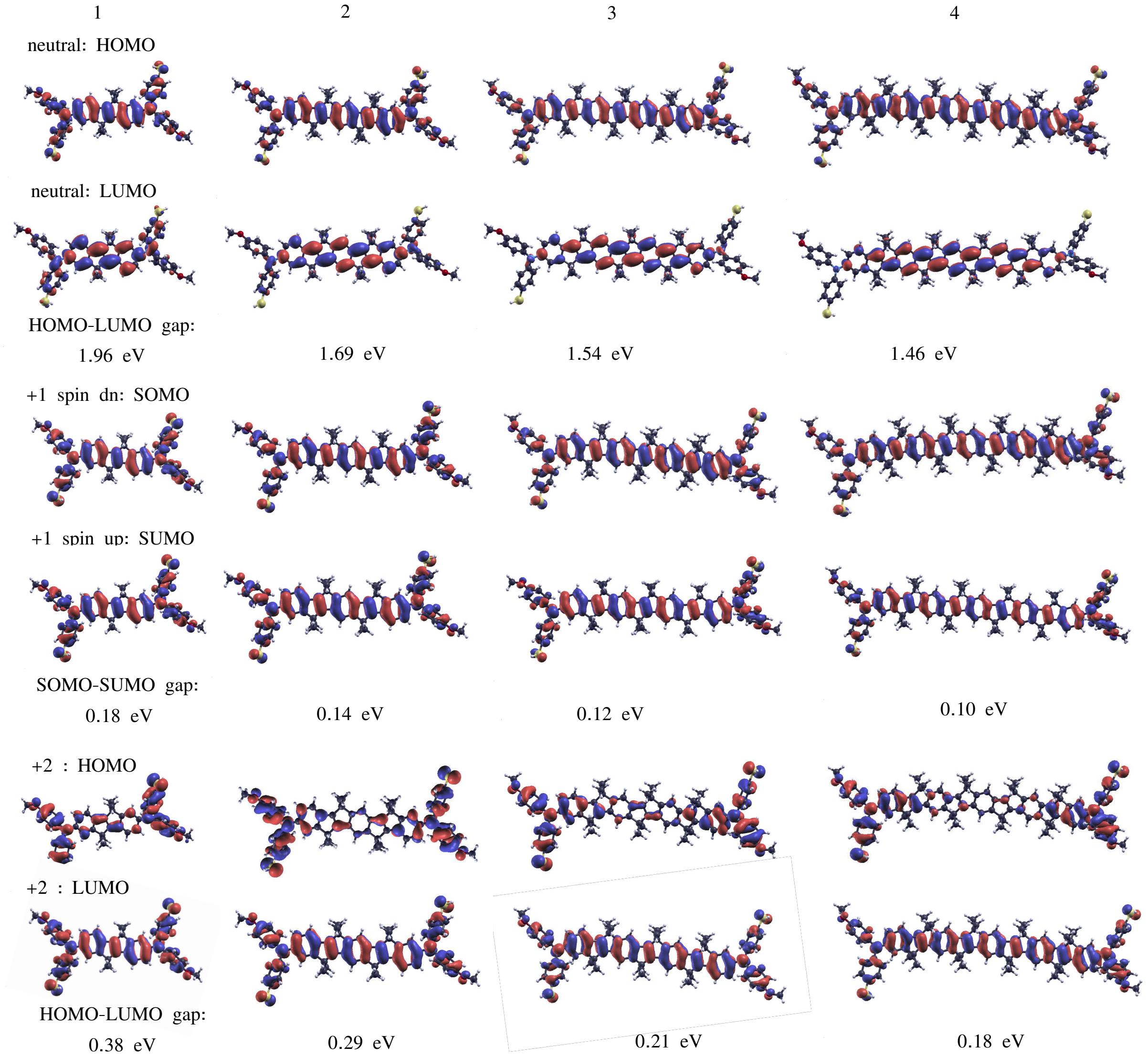

Figure S17: Delocalized molecular frontier orbitals of the isolated neutral and oxidized species of SMeDACOPV1-4.

6.3 Influence of Gold electrodes

We conducted zero-bias transport calculations of SMeDACOPV1 molecules using TranSiesta,[S17] modeling electrodes consisting of six layers of Au(111), with each layer containing a 6x6 arrangement of atoms. In the junction illustrated in Fig. S18 (a), the S atom is positioned at the face-centered cubic (fcc) site on the Au surface. For the geometry optimization, a k-grid of 2x2x2 was used and a mesh cutoff of 300 Ry. A SZ basis was used for Au, and a DZP basis for all other atoms. The molecular atoms were relaxed with a force tolerance of 0.02 V/Å. For the transport calculations, a k-grid of 3x3x1 was used and the same parameters otherwise, while for the transmission calculation, the k-grid was set to 6x6x1.

Figure S18 (b) presents a comparison of the transmission for two different electrode separations. Notably, we observe excellent agreement with the WBA results when the electrode distance is set to d = 2.6 Å. The presence of the H atom at site S did not play a significant role in these calculations.

To explore a similar junction, but with a different coupling to the gold electrodes, we focus on the configuration presented in Fig. S18 (c), where a single Au atom occupies the fcc position and forms a bond with the S atom. To increase the electrode-molecule interaction, we have removed the H atom from the S and decreased the electrode-electrode distance to d = 2.56 Å. This modification results in a significant shift of the HOMO and HOMO-1 peaks toward the Fermi level and to an enhancement of the transmission at E_F (Fig. S18 (d)). We ascribe this shift to an enhanced charge transfer from the electrode to the molecule in this scenario. In particular, a comparison of the electronic Mulliken population on the S atoms (see Table S3) reveals that in junction (a) there is less electron transfer to the S atoms than in junction (b).

Nevertheless, the peak widths and relative energetic positions of the frontier orbitals show minimal change. We anticipate that, even under these extreme circumstances, the trends identified in the main manuscript will remain reproducible.

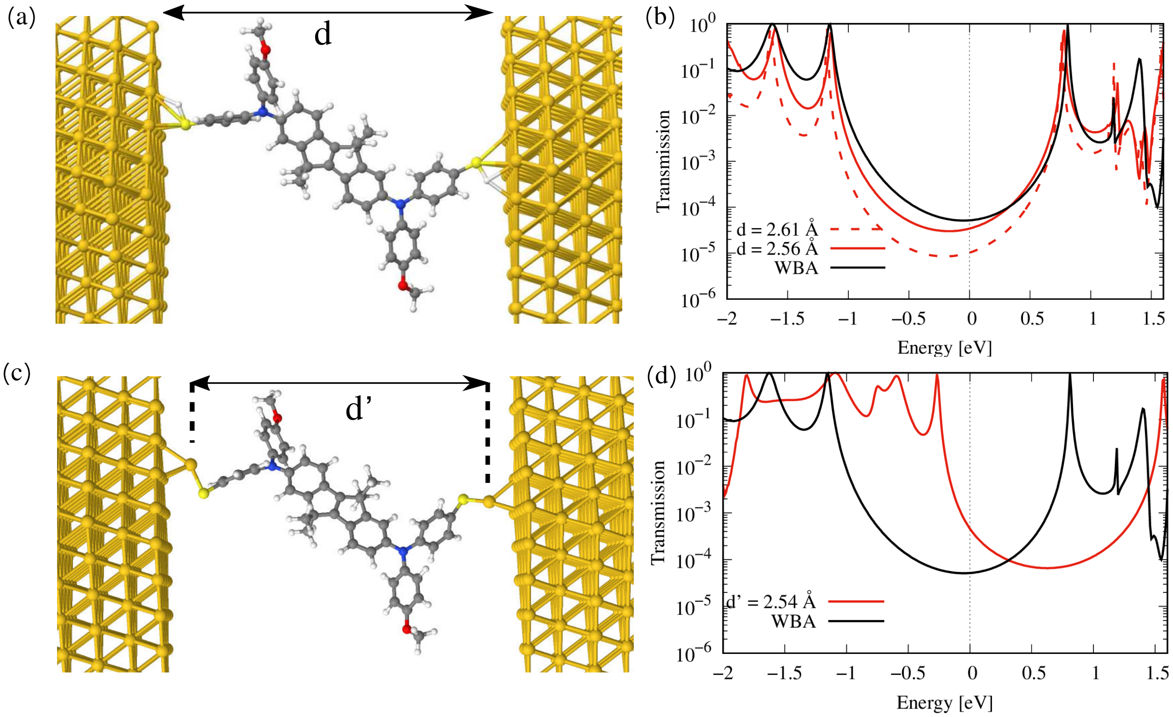

Figure S18: Neutral SMeDACOPV1 connected to gold electrodes. (a) The S atom is connected to the Au(111) electrode via the fcc hollow position, where d is the electrode-electrode distance. (b) Transmission through the junction shown in (a). At an electrode inter-distance of d = 2.56 Å, the transmission agrees very well with the WBA transmission (black line). (c) The molecule is connected to the Au(111) electrode via a single Au atom on the fcc hollow position. The Au-Au distance is d’. (d) The transmission through this junction is significantly shifted in energy, compared to the transmission in the WBA.

Table S3: Mulliken charge on S atom

S-atom	Pristine molecule	junction (a)	junction (b)
left	5.872 e	5.897 e	6.020 e
right	5.872 e	5.903 e	6.024 e

6.4 Influence of sidegroups

Figure S19 presents a comparison of the transmission properties of neutral SMeDACOPV2 and SMeDACOPV3. The results indicate that substituting the side groups with methyl groups has a negligible effect on transmission.

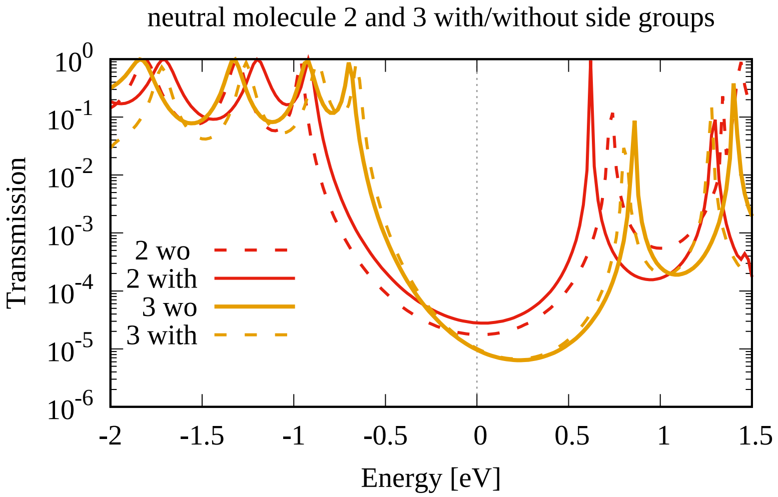

Figure S19: Transmission through neutral molecule SMeDACOPV2 and 3 with the real side groups and with methyl groups. The side groups have only minimal impact on transmission.

6.5 Influence of cis/trans configuration

Figure S20 presents a comparison of the transmission properties of the neutral and singly oxidized SMeDACOPV2 with the thioanisole moieties oriented towards the same or the opposite sides of the wire, which we denote 'cis' and 'trans' configuration (Fig. S20 A). For both orientations, the positions of the HOMO and LUMO peaks in the transmission are nearly the same. We find a trend for the cis configuration to result in a slightly lower transmission, which might be due to overall structural differences after the geometry optimization of the two conformers, however the difference in transmission is not significant.

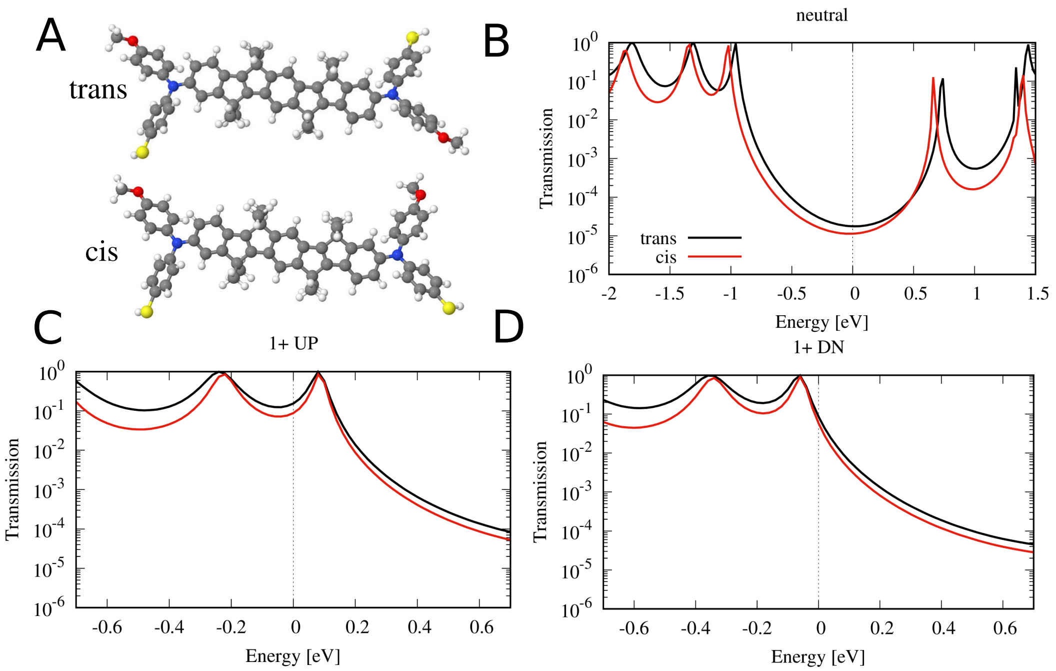

Figure S20: (A) Molecule SMeDACOPV2 in the cis and trans configuration with the respective transmissions through the neutral molecule (B) and the singly charged SMeDACOPV2 (C+D).

6.6 Analogy to topological states in periodic structures

To illustrate the analogy between SMeDACOPV and periodic topological insulators, we have calculated the band structure and density of states (DOS) of chains of SMeDACOPV. We achieved periodicity by linking the molecules with a carbon-carbon bond between the (formerly) sulfur-substituted groups (Fig. S21 a), and applied periodic boundary conditions using a k = (1, 1, 6) grid along the periodic direction.[S18] This approach enables us to calculate the band structure and DOS (Fig. S21 b,c), to identify the conducting states in the gap. For the charged system, we can identify topological states within the band gap, which arise from the nitrogen atoms acting as redox centers at the termini of the unit cell.

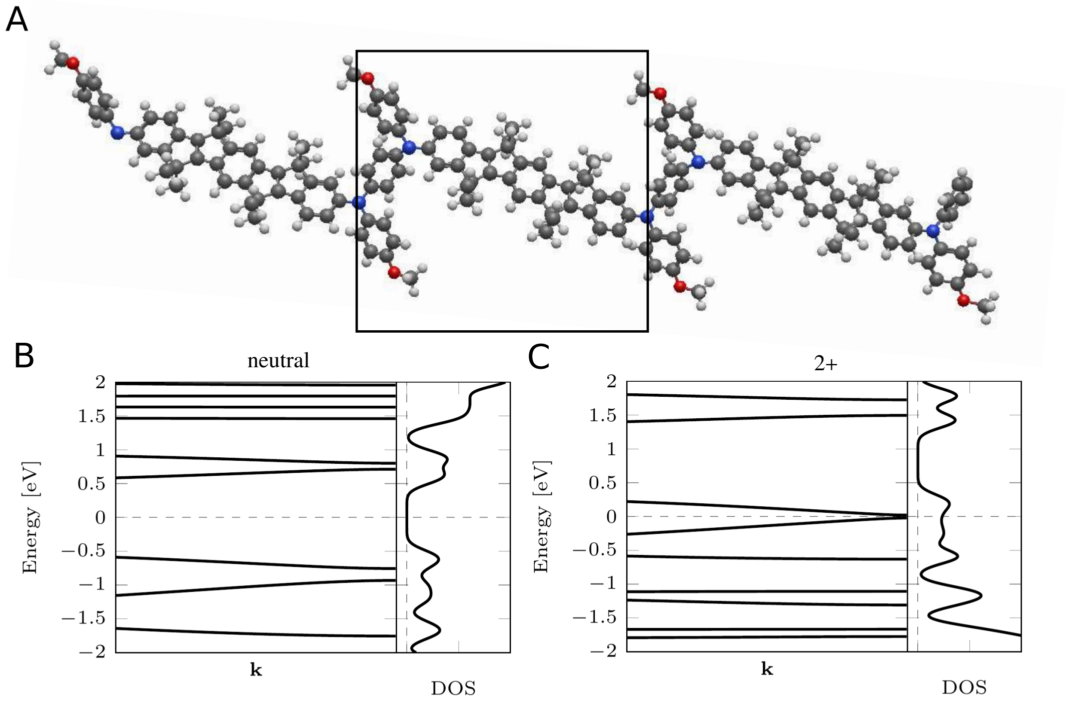

Figure S21: (A) Periodic wire built by SMeDACOPV2. The black box highlights the unit cell. (B) Band structure and density of states (DOS) of the neutral periodic SMeDACOPV2. The DOS in the gap is approximately zero. (C) Band structure and DOS of the dooubly oxidized periodic SMeDACOPV2-2+. There are two bands in the gap in close proximity to the Fermi energy, resulting in a finite DOS.

6.7 Spectral density of states in the junction

In the pristine molecules, where the electronic structure is represented by discrete enegetic levels rather than continuous energy bands, we have identified HOMO and LUMO for SMeDACOPVn^{+2} (SOMO and SUMO for SMeDACOPVn^{+1}) as highly conducting channels. For the molecule in the device, with electrodes described in the wide-band approximation, we can identify the corresponding peaks in the spectral DOS $A^{\alpha} = G(E)\Gamma_{\alpha}G^{\dagger}(E)$ for left and right going states ($\alpha = L, R$). This is shown in Figure S22 for SMeDACOPV2 and SMeDACOPV2^{2+}.

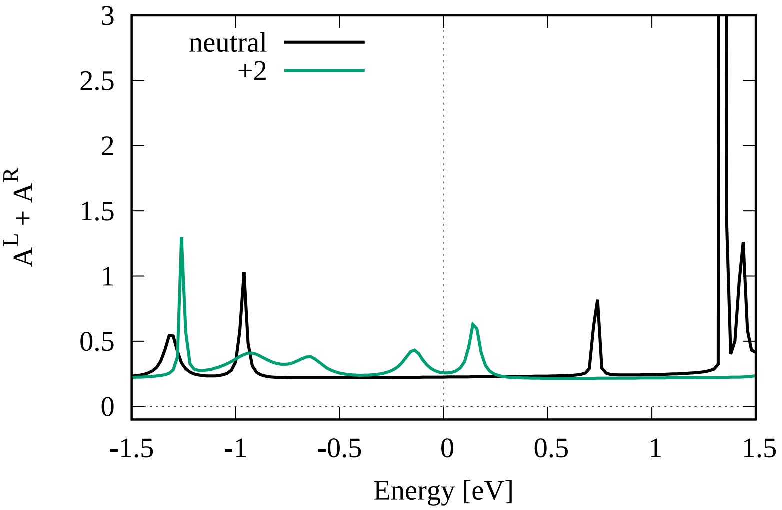

Figure S22: Spectral DOS $\sum_{\alpha=L,R} A^{\alpha}$ of SMeDACOPV2 and SMeDACOPV2^{+2}. For the doubly oxidized molecule, there are peaks at the energetic positions of HOMO and LUMO close to the Fermi level.

References

(S1) Zhu, X.; Tsuji, H.; López Navarrete, J. T.; Casado, J.; Nakamura, E. Carbon-bridged oligo(phenylenevinylene)s: Stable π-systems with high responsiveness to doping and excitation. *J. Am. Chem. Soc.* **2012**, *134*, 19254–19259.

(S2) Burrezo, P. M.; Lin, N.-T.; Nakabayashi, K.; Ohkoshi, S.-i.; Calzado, E. M.; Boj, P. G.; Díaz García, M. A.; Franco, C.; Rovira, C.; Veciana, J.; Moos, M.; Lambert, C.; López Navarrete, J. T.; Tsuji, H.; Nakamura, E.; Casado, J. Bis(aminoaryl) Carbon-Bridged Oligo(phenylenevinylene)s Expand the Limits of Electronic Couplings. *Angew. Chem. Int. Ed.* **2017**, *56*, 2898–2902.

(S3) Hussain, M. K.; Ansari, M. I.; Kant, R.; Hajela, K. Tandem C-2 Functionalization-Intramolecular Azide-Alkyne 1,3-dipolar Cycloaddition Reaction: A Convenient Route to Highly Diversified 9H-benzo [b]pyrrolo[1,2-g][1,2,3]triazolo[1,5-d][1,4]diazepines. *Org. Lett.* **2014**, *16*, 560–563.

(S4) Chen, X.; Li, M.; Liu, Z.; Yang, C.; Xie, H.; Hu, X.; Su, S. J.; Jiang, H.; Zeng, W. Bimetal Cooperatively Catalyzed Arylalkynylation of Alkynylsilanes. *Org. Lett.* **2021**, *23*, 6724–6728.

(S5) Cheng, X.; Ma, J.; Zhi, J.; Yang, X.; Hu, A. Synthesis of Novel "Rod-Coil" Brush Polymers with Conjugated Backbones through Bergman Cyclization. *Macromolecules* **2010**, *43*, 909–913.

(S6) Zhou, C.; Chen, X.; Lu, P.; Wang, Y. Synthesis of 2,3-diiodoindenes and their applications in construction of 13H-indeno[1,2-l]phenanthrenes. *Tetrahedron* **2012**, *68*, 2844–2850.

(S7) Zhu, X.; Tsuji, H.; Nakabayashi, K.; Ohkoshi, S. I.; Nakamura, E. Air- and heat-stable planar tri-p-quinodimethane with distinct biradical characteristics. *J. Am. Chem. Soc.* **2011**, *133*, 16342–16345.

(S8) Kodomari, M.; Satoh, H.; Yoshitomi, S. Selective halogenation of aromatic hydrocarbons with alumina-supported copper(II) halides. *J. Org. Chem.* **1988**, *53*, 2093–2094.

(S9) Xu, B.; Tao, N. J. Measurement of single-molecule resistance by repeated formation of molecular junctions. *Science* **2003**, *301*, 1221–1223.

(S10) Mang, A.; Rotthowe, N.; Beltako, K.; Linseis, M.; Pauly, F.; Winter, R. F. Single-molecule conductance studies on quasi-and metallaaromatic dibenzoylmethane coordination compounds and their aromatic analogs. *Nanoscale* **2023**, *15*, 5305.

(S11) Venkataraman, L.; Klare, J. E.; Nuckolls, C.; Hybertsen, M. S.; Steigerwald, M. L. Dependence of single-molecule junction conductance on molecular conformation. *Nature* **2006**, *442*, 904–907.

(S12) Venkataraman, L.; Klare, J. E.; Tam, I. W.; Nuckolls, C.; Hybertsen, M. S.; Steigerwald, M. L. Single-molecule circuits with well-defined molecular conductance. *Nano Letters* **2006**, *6*, 458–462.

(S13) Quek, S. Y.; Kamenetska, M.; Steigerwald, M. L.; Choi, H. J.; Louie, S. G.; Hybertsen, M. S.; Neaton, J. B.; Venkataraman, L. Mechanically controlled binary conductance switching of a single-molecule junction. *Nat. Nanotechnol.* **2009**, *4*, 230–234.

(S14) García, A.; Papior, N.; Akhtar, A.; Artacho, E.; Blum, V.; Bosoni, E.; Brandimarte, P.; Brandbyge, M.; Cerdá, J. I.; Corsetti, F.; Cuadrado, R.; Dikan, V.; Ferrer, J.; Gale, J.; García-Fernández, P.; García-Suárez, V. M.; García, S.; Huhs, G.; Illera, S.; Korytár, R.; Koval, P.; Lebedeva, I.; Lin, L.; López-Tarifa, P.; Mayo, S. G.; Mohr, S.; Ordejón, P.; Postnikov, A.; Pouillon, Y.; Pruneda, M.; Robles, R.; Sánchez-Portal, D.; Soler, J. M.; Ullah, R.; Yu, V. W.-z.; Junquera, J. Siesta: Recent developments and applications. *J. Chem. Phys.* **2020**, *152*, 204108.

(S15) Papior, N. sisl. 2023; `https://doi.org/10.5281/zenodo.597181`.

(S16) Soler, J. M.; Artacho, E.; Gale, J. D.; García, A.; Junquera, J.; Ordejón, P.; Sánchez-Portal, D. The SIESTA method for ab initio order-N materials simulation. *Journal of Physics: Condensed Matter* **2002**, *14*, 2745.

(S17) Papior, N.; Lorente, N.; Frederiksen, T.; García, A.; Brandbyge, M. Improvements on non-equilibrium and transport Green function techniques: The next-generation transiesta. *Comput. Phys. Commun.* **2017**, *212*, 8 – 24.

(S18) Li, L.; Louie, S.; Evans, A. M.; Meirzadeh, E.; Nuckolls, C.; Venkataraman, L. Topological Radical Pairs Produce Ultrahigh Conductance in Long Molecular Wires. *Journal of the American Chemical Society* **2023**, *145*, 2492–2498, PMID: 36689781.
